# Age-related differences in perceptual and mental imagery abilities

**DOI:** 10.3389/fpsyg.2025.1566776

**Published:** 2026-04-08

**Authors:** Anna Maria Berardi

**Affiliations:** 1 Cognitive Neuroscience Laboratory, Department of Psychology, Harvard University, Cambridge, MA, United States; 2 LCOMS, Département de Psychologie, UFR SHS-Metz, Université de Lorraine, Metz, Moselle, France

**Keywords:** dorsal ventral streams, visual perception, mental imagery, elderly, age

## Abstract

**Introduction:**

Previous studies have demonstrated the existence of two visual processing pathways in monkeys, healthy human participants, and patients with brain lesions. The dorsal and ventral visual pathways are involved in the perception of spatial locations and object recognition, respectively. Recent studies have challenged the idea that these two pathways are entirely segregated; however, their predominant roles in vision have not been questioned. The purpose of. this study was to investigate age-related differences in perceptual and mental imagery abilities.

**Methods:**

The Imagery Processing Battery (IPB), which includes 15 tasks for assessing different aspects of dorsal (spatial properties) and ventral (object properties) processing was used. Mean response times for each perceptual task were correlated with each other to obtain a correlation matrix; the corresponding correlation matrix was computed for the imagery tasks.

**Results:**

ANOVAs indicated that for the dorsal tasks, elderly participants were generally slower and made more errors than young participants, particularly in the imagery conditions. For the ventral tasks, older participants were slower but as accurate as young participants. These results remained unchanged when the simplest version of the generalized slowing hypothesis was tested using logarithmic response times. Nonmetric multidimensional scaling (MDS) of these correlation matrices in imagery and perception indicated the presence of two clusters, reflecting the dorsal/ventral nature of the tasks. However, the clusters based on data from the elderly were not as clearly defined as those based on data from the young.

**Discussion:**

These results suggest perceptual and mental imagery age-related changes in old as compared to young participants.

## 1 Introduction

The dissociation between the dorsal and ventral visual processing pathways was first characterized by Mishkin et al. (1983), and it is now widely recognized that these two streams play distinct roles in visual perception. Mishkin et al. (1983) taught monkeys to discriminate a particular shape or to choose a location to obtain a reward. Monkeys with bilateral lesions of the inferior temporal lobes had no problems remembering the location of the cue but were unable to discriminate its shape. In contrast, monkeys with bilateral lesions of the posterior parietal lobes discriminated the shape of the cue but could not use location as a cue. The pathway from the occipital lobe to the inferior temporal lobe has since been dubbed the “what” pathway, whereas the one from the occipital lobe to the posterior parietal lobe has been dubbed the “where” pathway (or sometimes the “how” pathway, emphasizing the role of spatial properties in motor control; Goodale and Milner, 1992; Whitwell et al., 2015; Ganel and Goodale, 2019).

The existence of these two pathways has been confirmed in healthy young human participants. Haxby et al. (1991) found that in young healthy participants, performance on a spatial task (determining the location of a dot) was associated with increases in regional cerebral blood flow, particularly in the parietal lobes. In contrast, performance on a ventral task (a face discrimination task) was associated with increases in regional cerebral blood flow in the inferior temporal lobes. Chen et al. (2000) found two behavioral clusters representing dorsal and ventral stream-dependent tasks in visual perception for young participants. Chen et al. (2002) further showed a less clear separation of dorsal and ventral stream-dependent tasks in visual perception for older participants.

Similarly, patients with brain lesions that selectively affect the dorsal or ventral visual processing streams display corresponding selective deficits in visual perceptual functions (Levine et al., 1985; Farah et al., 1988a,b). Selective deficits in dorsal and ventral visual processing have also been reported in mental imagery (Levine et al., 1985; Farah et al., 1988b; Luzzatti et al., 1998). Nevertheless, the overlap in the deficits of perceptual and mental imagery functions has not always been systematic, casting some doubt on the implications of the dorsal and ventral visual streams in both visual perception and mental imagery (e.g., Behrmann et al., 1994; Bartolomeo et al., 1997, 1998; Bartolomeo, 2008; Moro et al., 2008; Committeri et al., 2015; Spagna et al., 2021).

Brain imaging studies also generally support the distinction of the dorsal and ventral visual pathways in visual perception (Ungerleider and Haxby, 1994; Zeki et al., 1991; Haxby et al., 1991, 1993, 1994) and mental imagery (Kosslyn et al., 1993; Kosslyn, 1994; Kosslyn et al., 1998; D'Esposito et al., 1997; Mellet et al., 1996). However, functional brain imaging studies do not always provide support for the use of shared pathways in both visual perception and mental imagery (e.g., Kosslyn et al., 1999; Borst and Kosslyn, 2008; Ganis and Schendan, 2011; Cichy et al., 2012; Senden et al., 2019; Dijkstra et al., 2017, 2019; Kosslyn et al., 2001; Pearson et al., 2015; Pace et al., 2023). Notably, studies using fMRI with a Dynamic Causal Modeling (DCM) approach and Parametric Empirical Bayes (PEB) analyses to characterize the effective connectivity of BOLD responses demonstrate that, although there is significant overlap in the brain regions involved in visual perception and mental imagery, different network dynamics characterize these two functions. Indeed, it has been shown that the directional coupling and strength of brain regions involved in visual perception and mental imagery may differ (Mechelli et al., 2004; Sulpizio et al., 2020; Bencivenga et al., 2021; Tullo et al., 2022, 2023). Moreover, while both visual perception and mental imagery appear to rely on top-down processing, mental imagery relies exclusively on top-down processing, whereas visual perception also relies on bottom-up processing (Lee et al., 2012; Gilbert and Li, 2013; Dentico et al., 2014; Koenig-Robert and Pearson, 2021). Finally, the overlap of activated brain regions may not be entirely equivalent in visual perception and mental imagery, as some studies found no activation of area V1 during visual mental imagery (for reviews, see Kosslyn and Thompson, 2003; Pearson, 2019). However, the majority of studies did find activation of V1 during mental imagery (for a review, see Kosslyn and Thompson, 2003; Pearson et al., 2015; Pearson, 2019). Kosslyn et al. (1993, 1995) demonstrated the retinotopic activity of V1 in humans during mental imagery. Other studies highlight lesser activation of V1 during mental imagery compared to visual perception (e.g., Lee et al., 2012), with higher V1 activation associated with more vivid mental imagery (Cui et al., 2007; Lee et al., 2012; Dijkstra et al., 2017, 2019), or with the need to inspect details in high-resolution images (Kosslyn and Thompson, 2003), or with linking fine spatial features of mental objects so that these features can be preserved as a whole for further processing (“spatial binding”, Klein et al., 2004). However, the debate over the activation of area V1 in mental imagery appears to be resolved (Pearson, 2019), confirming the depictive view of mental imagery (Kosslyn, 1980, 1994) over the propositional view (Pylyshyn, 2002, 2003). We also demonstrated that in young healthy participants, the dissociation between the dorsal and ventral pathways can be observed at a purely behavioral level for a wide variety of equivalent visual perceptual and mental imagery tasks (Berardi, 2025).

Mental imagery is an important function involved in several high-level cognitive processes: executive functions, such as planning (Toussaint et al., 2013), and creative thinking (Palmiero et al., 2011; Pestry, 2023); mathematical ability, such as problem solving (Gros et al., 2025) or spatial navigation (Bird et al., 2012); language processing (e.g., vocabulary), as seen in reading, writing, and comprehension (Toskos Dils and Boroditsky, 2010; Commodari et al., 2020; Guarnera et al., 2019; Suggate and Martzog, 2020; Suggate and Lenhard, 2022; Williams and Suggate, 2024); and memory, such as in episodic or working memory (Keogh and Pearson, 2011, 2014; Pearson, 2019). The link between mental imagery and other cognitive functions has been best studied in children and adolescents, particularly in reading comprehension and language processing, as these functions are facilitated by the use of mental imagery in children as young as 4-5 years old (Commodari et al., 2020; Guarnera et al., 2019) and up to 10-13 years old in adolescents (Boerma et al., 2016; Commodary et al., 2024). Similar results have also been demonstrated in adults (Suggate and Lenhard, 2022). Interestingly, to assess the relation between mental imagery and cognitive function, Commodari et al. (2020) used the Mental Imagery Test (MIT) battery, which includes tests of image generation, inspection, and maintenance, specifically designed for use in children and adolescents (Di Nuovo et al., 2014). Given the important implications of mental imagery in other high-level cognitive processes, it is of interest to know whether mental imagery is preserved or affected in the elderly.

In the present study, we examined processing in the dorsal and ventral pathways in the elderly for both visual perception and mental imagery. Given that visuospatial processing decreases with age (see Hochandel and Kaplan, 1984; Klencklen et al., 2012; Techentin et al., 2014, but see also Laurin et al., 2023), it could be expected that old participants would display decreased performance on tasks relying on the dorsal pathway (Sciberras-Lim and Lambert, 2017). However, other studies have indicated that memory function is decreased in healthy old participants relative to young ones (e.g., Berardi et al., 1991, 1997). Therefore, old participants may also have greater difficulty with ventral processing tasks, which typically involve retrieving objects from long-term memory. Several studies have indicated that memory performance in elderly participants is affected by the type of material to be retrieved. Situations in which elderly participants have to retrieve from long-term memory stimuli that can be encoded as words and images (Paivio, 1969) show less impairment than those involving abstract verbal or unfamiliar stimuli (e.g., Berardi et al., 1991, 1997). Moreover, ventral tasks also rely on verbal mediation, a type of processing that is relatively preserved in healthy aging (Hochandel and Kaplan, 1984). Thus, previous research has not provided clear guidance regarding possible degradation in dorsal compared to ventral processing with aging.

However, there is some evidence suggesting age-related declines in performance on dorsal compared to ventral processing tasks. For example, Grady et al. (1994) found a more diffuse distribution of regional cerebral blood flow (rCBF) in older participants relative to young ones when they performed the Haxby et al. (1991) dorsal and ventral tasks. Grady et al. (1994) suggest that this finding could reflect the relative inefficiency of elderly participants in performing these tasks, as behavioral age-related deficits were found on both types of tasks. Several other studies have reproduced such findings in visual perception (Reuter-Lorenz et al., 1999; Grady et al., 2000; Cabeza, 2001, 2002; Voss et al., 2008; Reuter-Lorenz and Cappell, 2008; Grady, 2012; La et al., 2015; Srokova et al., 2020; Seider et al., 2021; Srokova et al., 2024), while others extended them to other cognitive functions in healthy aging, such as executive functions (Langenecker and Nielson, 2003; Langenecker et al., 2004), episodic memory (Morcom et al., 2003; Daselaar et al., 2003; Cabeza et al., 2004; Gutchess et al., 2005), and working memory (Park et al., 2003; Cabeza et al., 2004).

The aim of the present study was to determine whether there were age-related differences in visual perception and mental imagery in old relative to young participants. We expected to find decreased performance and slower response times on most visuospatial tasks (mediated by the dorsal pathway) in old relative to young participants. We also expected that old participants would be slower on all ventral tasks. This study not only evaluates perception but also the corresponding type of mental imagery. Mental imagery is generally a more complex process than visual perception, and therefore, more dramatic age-related changes are expected in this condition. Several of the visual perceptual and mental imagery processes reported in this study have not been evaluated previously in elderly participants.

## 2 Methods

### 2.1 Participants

Thirty-two elderly participants aged 60 to 73 were tested (mean 67, SD = 4). All but one participant had at least completed a college education. Half of the participants were Harvard alumni, while the other half had earned a college degree from other local universities (such as Boston University or MIT). The performance of the elderly participants was compared to that of 32 young healthy participants aged 17 to 22 (mean age 19, SD = 1), who were currently studying at Harvard University. Most of the young participants had not completed a bachelor's degree. Young participants were recruited through sign-up sheets posted on campus. Some of the old participants were recruited through the Harvard Alumni Record Office and had previously agreed to participate in cognitive studies. Since all participants in this study were highly educated, highly functioning individuals, we did not consider it necessary to screen them for cognitive impairment. For all participants, the testing session lasted 2.5 h, with a 10–15 min break after half of the tasks were administered. Preliminary screening regarding medications and other conditions that might affect brain function was completed during an initial telephone contact. During this preliminary screening, we asked all participants whether they had been previously diagnosed with or currently had any neurological, psychiatric, or medical conditions that would affect brain function or require medication. Any diagnoses of neurological, psychiatric, or medical conditions or medications that would affect brain function were exclusion criteria. All participants were healthy, as determined by a health questionnaire administered at the beginning of the study. Participants' reports indicated that they had no current psychiatric, neurological, or medical conditions that could affect brain function, confirming the information obtained during the initial telephone contact for all participants. Vision was normal or corrected to normal (20/25), and all participants wore glasses for the session if needed. All participants were right-handed, as determined by the Edinburgh Handedness Inventory (Oldfield, 1971). In both groups, half of the participants were males and half were females. This study was approved by the Harvard Ethical Committee and conducted in accordance with the principles of the Helsinki declaration. All procedures were described to participants, and written informed consent was obtained from all participants.

### 2.2 Measures

#### 2.2.1 Imagery Processing Battery (IPB)

The rationale behind the development of the Imagery Processing Battery (IPB) was to create an instrument to assess a wide variety of functions mediated by the dorsal and ventral visual pathways, using comparable tasks and equivalent levels of difficulty across visual perception and mental imagery. In this study, we used the IPB with young and old healthy participants to examine age-related differences in visual perceptual and mental imagery abilities. The tasks included in the IPB have been extensively described elsewhere (Berardi, 2025). Although all tasks likely recruit both types of processing, a key question is which type (dorsal or ventral) is most likely responsible for age-related differences. The ventral tasks were designed to predominantly engage ventral system processing. Therefore, differences in the efficacy of ventral processing should produce differences in our dependent measures (error rates and response times in the ventral tasks). Similarly, the dorsal tasks were designed to predominantly engage spatial processing. Therefore, differences in the efficacy of dorsal processing should produce differences in the related dependent measures (error rates and response times in the spatial tasks).

The Imagery Processing Battery (IPB) has been described in detail previously (Berardi, 2025). It includes 15 tasks that provide a comprehensive evaluation of comparable aspects of perceptual and mental imagery abilities. The tasks were divided into three major categories. The first category includes seven tasks that assess image generation (within grids and brackets), image rotation, image scanning, image maintenance, spatial imagery, and motor imagery, which are thought to be predominantly mediated by the dorsal pathway. The second category includes four tasks that require participants to evaluate the properties of common objects, such as auditory, color, face, and object imagery, which are thought to be predominantly mediated by the ventral pathway. The last category includes three tasks: word, size, and tactile imagery, which are believed to require mixed dorsal/ventral processing. In these three tasks, the dorsal/ventral distinction is subtle because representations of object properties need to be retrieved from long-term memory, but the judgment required is spatial. For a justification regarding why each task was classified as predominantly dorsal or ventral, for a more detailed description of the tasks, and examples of the stimuli used, see Berardi (2025).

#### 2.2.2 Apparatus

All tasks were implemented and administered on a Macintosh computer using the Superlab program (Cedrus Corporation, San Pedro, CA). Audacity and a stereo microphone were used to record sounds, and Paintbrush was used to rescale line drawings. An Epson Ecotank ET-7700 was used to digitize photographs, and Adobe Photoshop was used to crop and size them (Adobe Systems Inc., Mountain View, CA); if color balance needed adjustment, that was also accomplished with Adobe Photoshop. A maximum degree of brightness and contrast on the computer screen was used for all participants, and sounds were produced at a fixed volume for all participants.

#### 2.2.3 General procedure

All tasks included both a perceptual and an imagery condition and began with four practice trials, followed by 16 test trials (unless otherwise specified below). In the perception condition for the ventral tasks, participants typically saw pictures of objects on the computer screen, either individually or in pairs, and heard the names corresponding to the objects spoken by the computer in a male voice. Participants based their judgments on the pictures they saw, which disappeared only after a response key was pressed.

In the imagery conditions, participants heard only the names of the objects and based their judgments on their own mental images of the named objects. In both conditions, all items involved “close” discriminations: the items to be compared were not very different in the characteristic for which they needed to be judged, which has previously been shown to induce imagery (Chapter 9, Kosslyn, 1980; see also Berardi, 2025).

In the perception condition for the spatial tasks, participants typically studied the location of a set of squares and decided whether an “X” mark covered one of the stimuli, or whether an arrow pointed to a stimulus at a particular location. Participants based their judgments on the pictures they saw, which disappeared after a response key was pressed. In the imagery conditions, the stimuli were removed before the presentation of the “X” mark or arrow, and the participants visualized the stimulus to make their judgment.

Participants sat in a quiet room at a distance of 50 cm from the computer monitor.

For all tasks, instructions were presented on the computer screen and read by the participants. For all ventral tasks, two spatial tasks (image generation within grids and within brackets) and two mixed tasks (size and word imagery), there were two alternate and equivalent test versions. This was done to prevent participants from remembering the items or their judgments from the perceptual condition to the imagery one, or vice versa. The comparability of the two versions of each task was assessed based on ratings of similarity obtained from an independent group of 10–16 participants, depending on the task. For a more detailed description of how stimuli were chosen, see Berardi (2025). Whenever the tasks had two versions, half the participants received Version 1 in the perception condition and Version 2 in the imagery condition, while the other half received the opposite order; the order of conditions (perception, imagery) was counterbalanced across participants.

The response training task was administered to all participants at the beginning and end of the testing session. Participants then completed object imagery, image generation (brackets), word imagery, image rotation, tactile imagery, image scanning, size imagery, image generation (grids), auditory imagery, color imagery, image maintenance, spatial imagery, face imagery, and motor imagery tasks. Half the participants of each gender at each age received the order presented above, while the other half completed the tasks in reverse order. For all tasks and each condition (perception or imagery), half of the correct responses were “yes” and half were “no.” The trials were presented in a quasi-random order with the only restriction that “yes” and “no” responses never occurred more than three times in a row. The “yes” key was the letter “b” and the “no” key was the letter “n” on the computer keyboard. The keys were labeled “Y” for “yes” and “N” for “no.” All participants responded with their right hand and pressed the “Y” and “N” keys with their index and middle fingers, respectively. Participants advanced to the next stimulus by pressing the spacebar with the thumb of their dominant hand. They were instructed to respond *as* quickly *as* possible while remaining *as* accurate *as* possible.

Items in all tasks were “mini-blocked,” ensuring that each combination of manipulated variables occurred once before any combination could be repeated. Feedback was provided during the practice trials; the computer beeped if the incorrect response key was pressed. No feedback was provided during the test trials.

### 2.3 Statistical procedures

Response accuracy and response times were the dependent measures for all tasks. Mean response times and percent error were analyzed. Response times greater than two standard deviations from each participant's mean within a given cell, as well as response times for incorrect responses, were excluded from analyses. A maximum of 5% of the data were excluded for both young and old participants regarding error rates or response times in the perception or imagery conditions. Outliers were evenly distributed among young and old participants for error rates or response times in the perception or imagery conditions. There were no missing values in the means for any of the cells. Response times and error rates were analyzed using analyses of variance with one group factor (young vs. old participants) and, when appropriate, one within-group effect (complexity). Mean error rates and response times (±SD) for old participants are shown in Table 1a. The corresponding mean error rates and response times (±SD) for young participants are shown in Table 1b.

**Table 1a:** Mean error rates (%) and response times (ms) for IPB tasks in old participants.

**IPB tasks**		**Perception (mean** ±**SD)**		**Imagery (mean** ±**SD)**	
		**ER**	**RT**	**ER**	**RT**
**Response training**					
Session 1		3.62 ± 5.67	705 ± 156	**–**	**–**
Session 2		2.54 ± 4.73	661 ± 133	**–**	**–**
**Dorsal system tasks**					
Image generation – Brackets	Easy	0.39 ± 2.21	1,149 ± 335	5.47 ± 7.74	1,895 ± 545
	Difficult	1.17 ± 3.70	1,167 ± 309	17.58 ± 13.42	2,219 ± 714
	Near	0.39 ± 2.21	1,124 ± 275	7.03 ± 9.49	1,988 ± 695
	Far	1.17 ± 3.70	1,164 ± 329	16.02 ± 14.28	2,179 ± 567
Image generation – Grids	Easy	1.56 ± 5.27	1,197 ± 279	2.34 ± 4.96	1,889 ± 456
	Difficult	2.34 ± 5.89	1,253 ± 258	7.81 ± 10.41	2,628 ± 858
	Near	1.95 ± 7.18	1,225 ± 292	1.95 ± 5.60	2,084 ± 617
	Far	1.95 ± 5.60	1,232 ± 239	8.20 ± 9.84	2,428 ± 752
Image rotation	0°	1.56 ± 3.18	2,054 ± 620	**–**	**–**
	90°	**–**	**–**	15.63 ± 15.22	4,901 ± 2,068
	135°	**–**	**–**	16.02 ± 13.92	4,991 ± 2,079
Motor imagery	0°	2.15 ± 3.41	1,786 ± 860	**–**	**–**
	90°	**–**	**–**	4.30 ± 8.76	2,218 ± 6,83
	135°	**–**	**–**	3.13 ± 8.98	2,513 ± 812
Image scanning	Near	1.95 ± 5.60	1,151 ± 216	9.38 ± 11.88	1,140 ± 197
	Far	1.56 ± 4.20	1,286 ± 252	14.06 ± 13.75	1,251 ± 235
Image maintenance	2 squares	1.95 ± 4.61	1,111 ± 348	10.16 ± 10.74	1,587 ± 291
	4 squares	1.95 ± 4.61	1,128 ± 298	15.23 ± 14.80	2,110 ± 829
Spatial imagery	Short	5.47 ± 10.50	2,032 ± 564	13.28 ± 15.21	2,147 ± 713
	Long	6.25 ± 10.53	1,831 ± 598	12.11 ± 12.49	2,043 ± 594
**Ventral system tasks**					
Object imagery		5.66 ± 5.58	3,038 ± 348	9.57 ± 8.09	3,176 ± 360
Face imagery		12.30 ± 13.14	1,895 ± 683	28.32 ± 15.23	2,477 ± 717
Color imagery		14.65 ± 10.95	2,661 ± 574	25.39 ± 12.29	3,026 ± 706
Auditory imagery		26.17 ± 14.24	2,434 ± 755	30.47 ± 13.54	3,152 ± 1,016
**Mixed processing tasks**					
Word imagery		4.88 ± 6.49	1,403 ± 293	8.98 ± 8.24	2,341 ± 433
Size imagery		8.59 ± 12.06	1,541 ± 472	24.80 ± 10.82	3,118 ± 1,052
Tactile imagery		7.63 ± 13.49	2,008 ± 520	43.75 ± 14.01	3,145 ± 1,054

**Table 1b:** Mean error rates (%) and response times (ms) for IPB tasks in young participants.

**IPB tasks**		**Perception (mean** ±**SD)**		**Imagery (mean** ±**SD)**	
		**ER**	**RT**	**ER**	**RT**
**Response training**					
Session 1		3.13 ± 5.72	440 ± 66	–	–
Session 2		0.78 ± 2.10	441 ± 43	–	–
**Dorsal system tasks**					
Image generation – Brackets	Easy	1.56 ± 4.20	676 ± 158	1.17 ± 3.70	1,082 ± 239
	Difficult	2.73 ± 6.91	692 ± 219	8.59 ± 11.64	1,377 ± 461
	Near	0.78 ± 3.07	670 ± 157	2.34 ± 5.89	1,118 ± 255
	Far	3.52 ± 7.26	700 ± 218	7.42 ± 8.90	1,312 ± 413
Image generation – Grids	Easy	0.78 ± 3.07	689 ± 92	2.34 ± 4.96	1,191 ± 300
	Difficult	1.17 ± 3.70	735 ± 104	3.13 ± 7.10	1,436 ± 495
	Near	0.78 ± 3.07	706 ± 104	1.56 ± 4.20	1,123 ± 258
	Far	3.52 ± 7.26	714 ± 86	3.91 ± 6.69	1,305 ± 413
Image rotation	0°	2.23 ± 3.81	1,046 ± 215	–	–
	90°	–	–	9.77 ± 15.14	2,439 ± 677
	135°	–	–	8.98 ± 10.16	2,628 ± 825
Motor imagery	0°	2.34 ± 4.42	873 ± 194	–	–
	90°	–	–	2.34 ± 4.96	1,372 ± 325
	135°	–	–	2.73 ± 6.91	1,604 ± 501
Image scanning	Near	1.56 ± 4.20	767 ± 135	5.08 ± 10.93	690 ± 85
	Far	1.56 ± 4.20	826 ± 118	5.86 ± 11.87	738 ± 100
Image maintenance	2 squares	1.56 ± 4.20	717 ± 196	4.30 ± 6.82	1,120 ± 290
	4 squares	1.56 ± 5.27	723 ± 201	5.86 ± 10.03	1,323 ± 483
Spatial imagery	Short	4.69 ± 7.61	1,229 ± 230	8.20 ± 9.84	1,378 ± 442
	Long	3.13 ± 6.35	1,150 ± 241	8.20 ± 11.27	1,259 ± 350
**Ventral system tasks**					
Object imagery		6.06 ± 7.01	2,669 ± 217	8.59 ± 8.36	2,874 ± 306
Face imagery		1,055 ± 9.97	1,315 ± 512	23.05 ± 13.51	2,022 ± 525
Color imagery		9.18 ± 7.44	2,191 ± 388	25.78 ± 10.50	2,572 ± 436
Auditory imagery		13.09 ± 7.67	2,121 ± 625	23.63 ± 9.22	2,693 ± 947
**Mixed processing tasks**					
Word imagery		4.30 ± 4.88	941 ± 157	6.25 ± 8.55	1,758 ± 276
Size imagery		4.69 ± 9.91	1,028 ± 327	24.22 ± 12.48	2,322 ± 819
Tactile imagery		6.82 ± 15.91	1,583 ± 311	41.25 ± 13.26	2,126 ± 536

This table has been published originally in Berardi (2025).

We then used nonmetric Multidimensional Scaling (MDS) to determine whether there was less segregation of the two visual processing pathways in healthy old compared to young participants. Response times in the perceptual tasks were correlated with each other using Pearson correlations (pooling over levels of complexity), and the obtained correlation matrices were analyzed with MDS based on monotonicity coefficients (Shepard, 1962; Kruskal, 1964), with a maximum of 50 iterations to determine the spatial distribution of the IPB tasks. Nonmetric euclidian MDS were conducted separately for the perceptual and imagery data. Factor analysis was then used to obtain independent confirmation of the visual patterns identified with MDS (Harman, 1976). Multiple regression analyses were implemented to determine which attribute(s) best accounted for the two MDS dimensions in perception and imagery. All statistical analyses were carried out using SPSS (Armonk, NY: IBM Corp.).

## 3 Specific methods, procedures, and Results

### 3.1 Response training

The purpose of this task was to familiarize participants with the general procedure used for all tasks in the IPB. By comparing the results from the response training task when it was administered at the beginning with when it was administered at the end of testing, we could detect changes related to practice or fatigue effects.

#### 3.1.1 Method

##### 3.1.1.1 Materials

The word “yes” or “no” appeared in uppercase letters at the center of the computer screen. Words subtended a visual angle of 2.3° horizontally and 1.2° vertically and were presented in black ink on a white background. Each word appeared eight times.

##### 3.1.1.2 Procedure

An exclamation point appeared at the center of the computer screen. The participants were instructed to focus on it and then to press the space bar when they were ready to see the next stimulus. After 500 ms, the word “yes” or “no” appeared, and participants pressed the “Y” key if the word was “yes” and the “N” key if the word was “no.” Each word remained on the screen until a response key was pressed. Immediately thereafter, the exclamation point returned, and a new trial began.

##### 3.1.1.3 Results

###### 3.1.1.3.1 Error rates

Young and old participants made equivalent numbers of errors on the first administration of the task (see Tables 1a, b), *F* < 1. On the second administration, however, older participants tended to make more errors than the young, *F*_(l, 62)_ = 3.70, *p* = 0.06.

###### 3.1.1.3.2 Response times.

Old participants were slower than young participants on the first, *F*_(1, 62)_ = 78.22, *p* = 0.0001, and on the second administration of the task, *F*_(1, 62)_ = 79.08, *p* = 0.0001.

In summary, old participants were slower than the young on both the first and second administrations of the task, indicating that any deficits in overall response times observed in other IPB tasks may be related to differences in low-level encoding or response processes.

### 3.2 Image generation: brackets

Variations in the response times and error rates from the following seven tasks should predominantly reflect the efficacy of processing in the dorsal system. Image generation is the process by which visual images are formed based on information stored in long-term memory. This process requires activating stored visual information to recreate a spatial pattern. The image generation task, based on one originally designed by Podgorny and Shepard (1978), has been used in modified versions by Kosslyn and collaborators (e.g., Dror et al., 1993; Dror and Kosslyn, 1994; Kosslyn et al., 1993, 1995). In the brackets version of the image generation task, participants decide whether an “X” covers an uppercase letter, either while the letter is present or while participants visualize it. When participants must decide whether an “X” covers a letter in imagery, they take more time and make more errors when the “X” is placed on a segment that is typically drawn later in the sequence of strokes than when it is placed on a segment drawn earlier. They also make more errors and are slower when the letters are complex (i.e., when they are composed of four or five segments as opposed to 3; see Kosslyn et al., 1988). In perception, response times and error rates are not affected by letter complexity or probe location (e.g., Kosslyn et al., 1988). We expected old participants to be slower and make more errors than young participants on this spatial task. One previous study examined age differences on an imagery version of this task (Dror and Kosslyn, 1994) and found that old individuals were more affected by increasing complexity than young individuals, but not by probe distance (for both error rates and response times); moreover, old participants were generally slower and less accurate than young participants.

#### 3.2.1 Method

##### 3.2.1.1 Materials

Uppercase letters (F, G, C, and P) were presented in shaded gray at the center of the computer screen within four brackets forming the corners of an imaginary 4 × 5 grid. Two of the letters (F, C) had 3 segments (simple condition) and two (P, G) had 4 and 5 segments, respectively (complex condition). Letters subtended a visual angle of 3° horizontally and 3.6° vertically. In half of the trials, the “X” covered the letter, while in the other half it did not. In half of the trials, the “X” appeared on an “early” segment or adjacent to it, and in the other half on a “late” segment or adjacent to it. If the probe was on the letter (or would have been on the letter in the imagery condition), the response was “yes”; if it was adjacent to it (or would have been), the response was “no.” Two comparable versions of this task were created, in which the “X” appeared at different on/off positions. This was done to prevent the participants from remembering the position of the “X” from the previous condition.

##### 3.2.1.2 Procedure

This task began with a learning phase in which participants familiarized themselves with the letters to be used in the experiment. Participants saw an exclamation point at the center of the computer screen, and 400 ms after pressing the spacebar, a lowercase letter was presented centrally below the four brackets, while the uppercase version of the same letter was displayed within the brackets. Participants studied the uppercase version of each letter. They then pressed the spacebar, and 400 ms later saw another letter.

After all letters were presented three times each, in a random order, with the only constraint that all letters had to be presented once before any letter would be repeated, participants drew the letters from memory within empty sets of brackets printed on sheets of paper. When participants pressed the spacebar, the lowercase version of a letter appeared at the center of the screen. Participants drew the corresponding uppercase version of the letter on the paper as accurately as possible without time constraints. Once they finished drawing one letter, they pressed the spacebar, and another letter was presented.

If all uppercase letters were drawn correctly, the experimental task was conducted. If not, participants were asked to study the letters again, following the same procedure. If all the letters were correctly drawn, participants proceeded to the next part of the experiment. If not, they received a final practice session in drawing the letters, as previously described. All young and elderly participants in this experiment were able to draw all the letters correctly on the first try.

In the perception condition, an exclamation point appeared 500 ms after the participant pressed the spacebar. A lowercase letter from the studied set was displayed for 500 ms at the center of the computer screen, and 500 ms later, it was replaced by a lowercase letter presented below a set of four brackets. At the same time, the uppercase version of the letter appeared within the brackets, along with an “X” at a specific location within the brackets. Participants decided whether the “X” covered the uppercase version of the letter. If the “X” covered the letter, participants pressed the “Y” key; if it did not, they pressed the “N” key. After a response key was pressed, the exclamation point returned to the screen.

In the imagery condition, participants were presented only with the lowercase letter for 500 ms. After 500 ms, an “X” appeared within the brackets, but the uppercase version of the letter was not present. Participants visualized the uppercase version of the letter within the brackets and decided whether an “X” would cover the letter if it were present.

##### 3.2.1.3 Results

###### 3.2.1.3.1 Error rates

In the perception condition, old participants made marginally more errors than young participants, *F*_(1, 62)_ = 3.23, *p* = 0.08 (with means of 2% and 1%). As expected, the complexity effect was not significant, *F*_(1, 62)_ = 1.30, *p* = 0.26. All participants also made more errors with late than early probes, *F*_(1, 62)_ = 4.52, *p* = 0.04 (with means of 2% and 1%). However, old participants did not make disproportionately more errors than young participants with late relative to early probes, *F*_(1, 62)_ = 1.40, *p* = 0.24.

In the imagery condition, old participants made more errors than young participants, *F*_(1, 62)_ = 12.49, *p* = 0.0008 (with means of 12% and 5%). As expected, in the imagery condition, all participants made more errors with complex than with simple letters, *F*_(1, 62)_ = 37.44, *p* = 0.0001 (13% vs. 3%, respectively). No other main effects or interactions were significant. Therefore, old participants did not make disproportionately more errors for complex relative to simple letters compared to young participants.

In the imagery condition, all participants made more errors with late than early probes, *F*_(1, 62)_ = 17.39, *p* = 0.0001 (12% and 5%). No other main effects or interactions were significant. Therefore, old participants did not make disproportionately more errors with late than early probes relative to young participants.

###### 3.2.1.3.2 Response times

In visual perception, old participants were overall slower than young participants, *F*_(1, 62)_ = 54.50, *p* = 0.0001 (with means of 1,158 and 684 ms). As expected, all participants had equivalent response times for complex relative to simple letters, *F*_(1, 62)_ = 1.08, *p* = 0.30 (with means of 929 and 913 ms). All participants were also marginally slower in the perception condition with late than early probes, *F*_(1, 62)_ = 3.95, *p* = 0.051 (with means of 932 and 897 ms). The complexity × group and the probe distance × group interactions were not significant. Therefore, as expected in perception, old participants did not make disproportionately more errors than young participants with complex relative to simple letters.

In mental imagery, old participants were overall slower than young participants, *F*_(1, 62)_ = 49.67, *p* = 0.0001 (with means of 2,057 and 1,230 ms). As expected, all participants were slower with complex than simple letters in the imagery condition, *F*_(1, 62)_ = 31.67, *p* = 0.0001 (with means of 1,798 and 1,489 ms). All participants were also slower with late than early probes, *F*_(1, 62)_ = 9.98, *p* = 0.002 (with means of 1,746 and 1,553 ms). The complexity × group and the probe distance × group interactions were not significant.

As expected, we found a significant effect of complexity and probe distance in the imagery condition, but not in the perception condition, for all participants, replicating previous findings (e.g., Kosslyn et al., 1988). We also found that old participants tended to make more errors than the young in the perception condition, made more errors than the young in the imagery condition, and were overall slower than the young in both conditions. In contrast to the findings of Dror and Kosslyn (1994), the age × complexity and the age × probe distance interactions were not significant in the imagery condition, suggesting that the elderly participants were as efficient as the young participants at generating mental images and were not affected by letter complexity or probe distance in their decisions.

### 3.3 Image generation: grids

A variation of the image generation task has been shown previously to recruit more left-hemisphere processing, as opposed to the right-hemisphere processes used in the previous version (Kosslyn et al., 1995).

#### 3.3.1 Method

##### 3.3.1.1 Materials

Participants studied uppercase versions of 4 letters within a 4 × 5 grid rather than within brackets. Different letters were used than in the brackets task. Two letters had 3 segments (H, U; simple condition) and two had 4 and 5 segments, respectively (J, S; complex condition). Stimuli subtended a visual angle of 3° horizontally and 3.6° vertically. In half the trials, the “X” would cover the letter; in the other half, it would not. In half the trials, the “X” appeared on a “near” segment, while in the other half, it appeared on a “far” segment. Half the letters were simple, and half were complex. There were two comparable versions of this task, where the “X” appeared at different on/off positions, to prevent participants from remembering the position of the “X” from the previous condition.

##### 3.3.1.2 Procedure

The experimental procedure for this task was identical to that described for the brackets generation task.

##### 3.3.1.3 Results

###### 3.3.1.3.1 Error rates

In the perception condition, no significant results were found.

**Figure 1:**
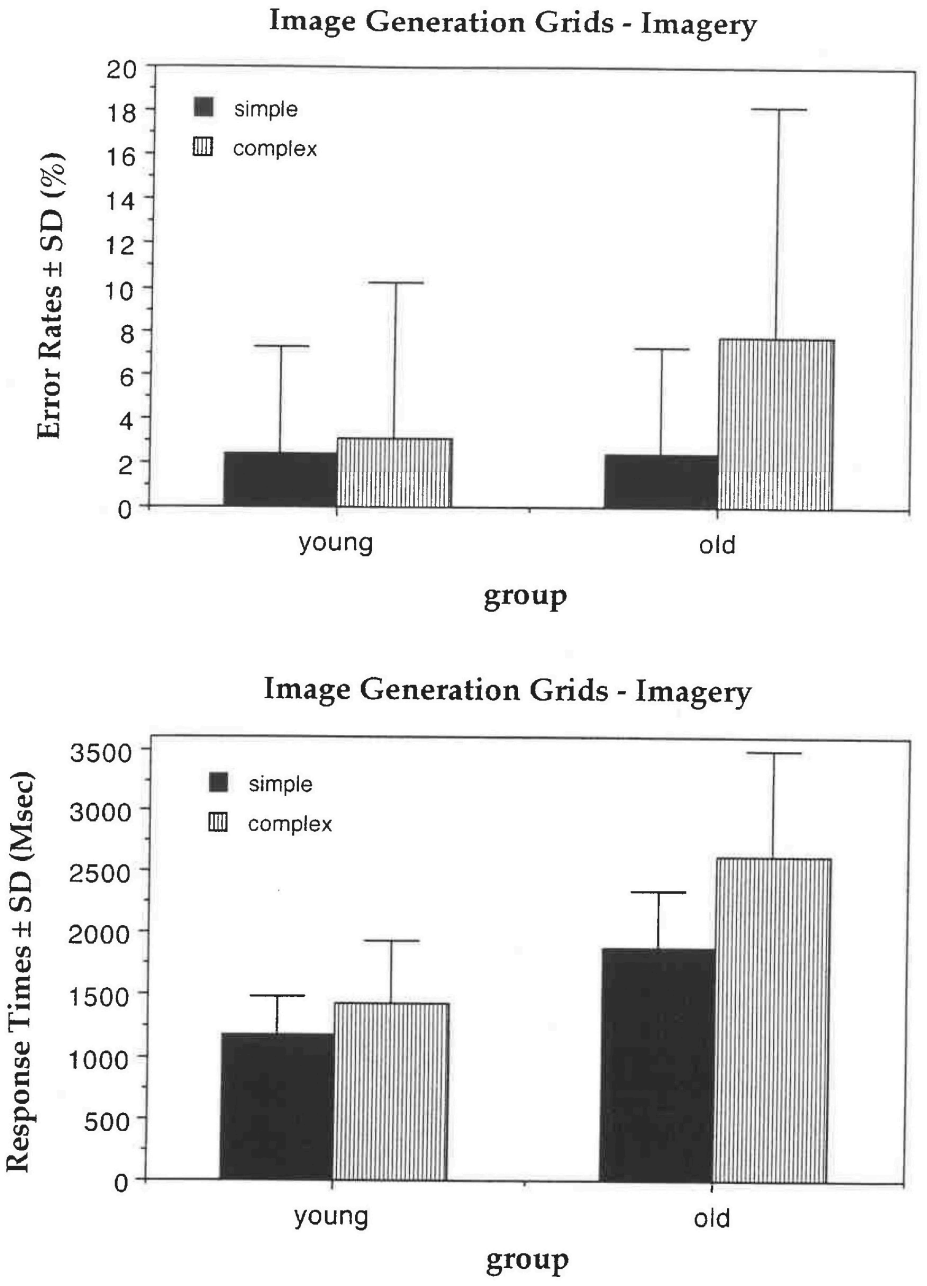
Image generation grids task. In the imagery condition, old participants tended to make more errors and were disproportionately slowed relative to the young with the complex compared to the easy letters.

In the imagery condition, old participants showed a tendency to make more errors than young participants, *F*_(1, 62)_ = 3.60, *p* = 0.06 (with means of 3% and 5%). For all participants, the complexity effect was significant, *F*_(1, 62)_ = 5.67, *p* = 0.02 (with means of 2% and 5%), as was the probe distance effect, *F*_(1, 62)_ = 12.72, *p* = 0.0007 (with means of 2% and 6%). Old participants tended to make more errors with complex letters than with simple letters compared to young participants, *F*_(1, 62)_ = 3.19, *p* = 0.08 (Figure 1), suggesting a tendency for an age-related decline in image generation within grids. No other main effects or interactions were significant.

###### 3.3.1.3.2 Response times

In the perception condition, old participants were overall slower than young participants, *F*_(1, 62)_ = 112.76, *p* = 0.0001 (with means of 1,225 and 712 ms). For all participants, response times were slower with more complex letters, *F*_(1, 62)_ = 11.37, *p* = 0.001 (with means of 994 and 943 ms). No other main effects or interactions were significant.

In the imagery condition, old participants were overall slower than young participants, *F*_(1, 62)_ = 54.26, *p* = 0.0001 (with means of 2,259 and 1,314 ms). For all participants, response times were slower with complex letters compared to simple letters, *F*_(1, 62)_ = 68.65, *p* = 0.0001 (with means of 2,032 and 1,540 ms). Old participants were also disproportionately slower compared to young participants with complex letters vs. simple letters, *F*_(1, 62)_ = 17.30, *p* = 0.0001 (Figure 1). Finally, old participants were slower with late probes compared to early probes, *F*_(1, 62)_ = 17.04, *p* = 0.0001 (with means of 1,866 and 1,604 ms), suggesting an age-related decline in image generation within grids.

In summary, in the grids version of the image generation task, old participants tended to make more errors overall than young participants in the imagery condition, although in the perception condition their error rates were comparable. Moreover, in the imagery condition, old participants were slower and tended to make more errors than young participants with complex letters compared to simple letters. Surprisingly, letter complexity, but not probe distance, also affected response times in the perception condition. It is likely that, to locate the probe, participants followed the drawing sequence of the segments composing the letter, although the letter was physically present.

### 3.4 Image rotation

This task assesses the ability to transform visual mental images. It is a modification of one originally developed by Shepard and Metzler (1971) and Shepard and Cooper (1982), who showed that response times and error rates increase linearly with greater angular disparities (Shepard and Cooper, 1982). We expected our elderly participants to show age-related decrements in performance on this spatial task. Dror and Kosslyn (1994) evaluated the imagery condition of this task and found that elderly participants were slower and made more errors than young participants when rotating images with greater angular disparities.

#### 3.4.1 Method

##### 3.4.1.1 Materials

Participants viewed two patterns, one on the left and one on the right of a central fixation point. The patterns were composed of five squares, each connected to another on at least one side. The pattern on the right of the central fixation was either identical to or a mirror image of the pattern on the left. The top of each pattern was indicated by a square filled with black. Participants were instructed to determine whether the shape on the right was the same as the shape on the left. The shapes were considered identical if the shape on the right could be moved to perfectly cover the shape on the left. They were deemed not identical if the shape on the right had to be flipped over before perfectly covering the shape on the left. Participants pressed the “Y” key if the two patterns were identical and the “N” key if they were not. The stimuli subtended a maximum visual angle of 5° horizontally and 3.6° vertically and were presented 1.5° to the right or left of the central fixation point. The pattern on the right was rotated at 0° (in the perception condition) or 90° and 135° (in the imagery condition). Degree of rotation and type of response were counterbalanced in both conditions.

##### 3.4.1.2 Procedure

In the perception condition, an exclamation point appeared on the screen until participants pressed the spacebar. After 500 ms, a centered star was presented, followed 500 ms later by a pattern appearing to the left and another to the right of the fixation point. Participants decided whether the pattern on the right was the same as the pattern on the left.

In the imagery condition, an exclamation point was presented until participants pressed the spacebar, and 500 ms later, a centered star appeared, followed after another 500 ms by two patterns. The pattern on the left was always presented upright (with the black square on top). The pattern on the right was rotated either 90° or 135°. Participants decided whether the pattern on the right was the same as the pattern on the left.

##### 3.4.1.3 Results

###### 3.4.1.3.1 Error rates

In the perception condition, error rates were identical for old and young participants (2%, *F* < 1). No other main effects or interactions were significant.

In the imagery condition, old participants made more errors overall than the young participants, *F*_(1, 62)_ = 4.29, *p* = 0.04 (with means of 16% and 9%, respectively). However, the angle × age interaction was not significant (with means of 10% and 9% for 90° rotations and 16% for both age groups in 135° rotations), *F* < 1. No other main effects or interactions were significant.

###### 3.4.1.3.2 Response times

In the perception condition, old participants were overall slower than the young participants, *F*_(1, 62)_ = 75.44, *p* = 0.0001. No other main effects or interactions were significant.

In the imagery condition, old participants were overall slower than young participants, *F*_(1, 62)_ = 46.08, *p* = 0.0001. The angle × age interaction was not significant (with means of 2,439 and 2,628 ms for 90° rotations and 4,901 ms and 4,991 ms for 135° rotations in young and old participants, respectively), *F* < 1. No other main effects or interactions were significant.

When we considered the 0°, 90°, and 135° rotations in a single analysis, we obtained the following results:

###### 3.4.1.3.3 Error rates

The overall group effect was marginally significant, with old participants making more errors than the young (with means of 11% and 7%), *F*_(1, 62)_ = 3.70, *p* = 0.06. All participants made more errors with greater angular disparities (with means of 2%, 13%, and 13% for 0°, 90°, and 135°, respectively), *F*_(2, 124)_ = 26.87, *p* = 0.0001.

The age × angle interaction was significant: old participants made more errors than the young with greater angular disparities (with means of 2%, 16%, and 16% for old participants and 2%, 10%, and 9% for young participants for 0°, 90°, and 135° rotations, respectively), *F*_(2, 124)_ = 3.16, *p* = 0.046, indicating that the elderly had more difficulty in image transformation than the young.

###### 3.4.1.3.4 Response times

Old participants were overall slower than the young (with means of 3,982 ms and 2,038 ms), *F*_(1, 62)_ = 52.73, *p* = 0.0001. All participants were slower with greater angular disparities (with means of 1,550, 3,670, and 3,809 ms for 0°, 90°, and 135°, respectively), *F*_(2, 124)_ = 124.90, *p* = 0.0001. The age × angle interaction was significant, indicating that old participants were slower than the young with greater angular disparities (with means of 2,054, 4,901, and 4,991 ms for old participants and 1,046, 2,439, and 2,628 ms for young participants for the 0°, 90°, and 135° rotations, respectively), *F*_(2, 124)_ = 12.87, *p* = 0.0001, indicating an age-related change in image transformation.

In summary, when we analyzed the perceptual and imagery conditions separately, our results contrast with those of Dror and Kosslyn (1994). They found a significant age × angular disparity interaction for both error rates and response times. Both interactions were not significant in our study. However, when we included the 0° rotation in the analyses, as was done by Dror and Kosslyn (1994), the angular disparity interaction became significant, replicating their results for both error rates and response times.

### 3.5 Motor imagery

This task is similar to the image rotation task, but hands are used as stimuli instead of connected sets of squares. Shepard and Cooper (1982) showed that when participants are required to rotate hands, they usually imagine rotating their own hands (see also Parsons, 1987).

#### 3.5.1 Method

##### 3.5.1.1 Materials

Stimuli were hands similar to those originally used by Shepard and Cooper (1982); they consisted of either two right hands or two left hands (“yes” responses), or one right and one left hand (“no” responses). Participants saw either two palms or two backs of the hands. Stimuli subtended a visual angle of 3° both horizontally and vertically and were displaced 1° to the right and left of a central fixation point. The hand on the right was rotated 0° (in perception) or 90° to 135° (in imagery). Degree of rotation, yes/no responses, palm or back of the hands, and right/left hands were all counterbalanced.

##### 3.5.1.2 Procedure

The procedure was the same as for the image rotation task. This time, participants were instructed that the “standard” position of the hands was with the fingers at the top.

##### 3.5.1.3 Results

###### 3.5.1.3.1 Error rates

In the perception condition, no main effects or interactions were significant.

In the imagery condition, no main effects or interactions were significant.

###### 3.5.1.3.2 Response times

In the perception condition, old participants were slower than the young participants, *F*_(1, 62)_ = 34.38, *p* = 0.0001 (with means of 1,786 and 873 ms). No other main effects or interactions were found.

In the imagery condition, old participants were slower than the young participants, *F*_(1, 62)_ = 36.66, *p* = 0.0001 (with means of 2,365 and 1,488 ms). As expected, in the imagery condition all participants were slower when the shapes were rotated 135° compared to 90°, *F*_(1, 62)_ = 32.39, *p* = 0.0001 (with means of 1,795 and 2,058 ms). However, there was no interaction between angle and age, *F* < 1.

We then included in the analyses the 0° rotation, along with the 90° and 135° rotations.

###### 3.5.1.3.3 Error rates

The main age effect and the age × angular disparity interaction were not significant, both *F* < 1. The number of errors did not differ as a function of angular disparity, *F* < 1.

###### 3.5.1.3.4 Response times

Old participants took longer than the young participants (with means of 2,172 and 1,283 ms, respectively), *F*_(1, 62)_ = 43.08, *p* = 0.0001. All participants took longer with greater degrees of angular disparity (with means of 1,329, 1,795, and 2,058 ms for 0°, 90°, and 135° rotations), *F*_(2, 124)_ = 70.42, *p* = 0.0001. However, the age × angular disparity interaction was not significant, *F* < 1.

Thus, in this task, we have evidence that participants used imagery; however, the age × angular disparity interaction was not significant. These results contrast with those from the mental rotation task. Apparently, the elderly have an age-related change in rotating arbitrary objects but not in rotating hands.

### 3.6 Image scanning

Image scanning is the ability to shift attention to different parts of a visualized object. Based on a task developed by Finke and Pinker (1982), which was modified by Kosslyn et al. (1990) and Dror et al. (1993) as well as Dror and Kosslyn (1994), the perceptual condition of this task involves the presentation of a donut-like square grid formed by smaller squares, three of which are black. Participants decide whether an arrow points to the center of a black square. In the imagery condition, the square grid is presented very briefly, and participants are instructed to scan a mental image to determine whether the arrow would have pointed to one of the black squares if the grid were still present. Participants typically take longer and make more errors when they scan greater distances (Denis and Kosslyn, 1999a,b; Dror et al., 1993; Dror and Kosslyn, 1994; Kosslyn, 1973; Kosslyn et al., 1978). Effects of aging on this task have been reported in some studies (Brown et al., 1998) but not in others (Dror and Kosslyn, 1994; see also Folk and Hoyer, 1992).

#### 3.6.1 Method

##### 3.6.1.1 Materials

A donut-shaped square grid was presented at the center of the computer monitor and subtended a visual angle of 7° both horizontally and vertically. The grid was composed of smaller squares, and three randomly selected squares within this grid were black. The locations of the black squares, the directions of the arrows (north, south, east, or west), and the positions of the arrows (right, left, top, and bottom) were counterbalanced. The arrows appeared at one of two possible distances from the target: near arrows were located 0.5 cm from the inside border of the grid, and far arrows were located 2 cm from the inside border of the grid. Half of the arrows were near, and half were far; in each case, half the time the arrow pointed to a square that was black, and half the time it did not.

##### 3.6.1.2 Procedure

In the perception condition, 500 ms after participants pressed the spacebar, a donut-shaped square grid appeared at the center of the computer monitor. Participants memorized the locations of the three black squares and then pressed the spacebar; 250 ms later, an arrow appeared within the donut-shaped square grid and remained visible until a response key was pressed. Participants pressed the “yes” key if the arrow pointed to the center of one of the black squares, and the “no” key if it did not.

In the imagery condition, participants studied the locations of the black squares, but this time after they pressed the spacebar, an arrow was flashed for only 50 ms within the grid, at which point both the arrow and the grid disappeared. Participants scanned their mental images to decide whether the arrow was pointing to the center of one of the black squares.

##### 3.6.1.3 Results

###### 3.6.1.3.1 Error rates

In the perception condition, no main effects or interactions were significant.

In the imagery condition, old participants made more errors overall than the young, *F*_(1, 62)_ = 6.09, *p* = 0.02 (12% and 5%). All participants also had a slight tendency to make more errors with far arrows compared to close arrows, *F*_(1, 62)_ = 2.66, *p* = 0.108 (means of 10% and 7%). No other main effects or interactions were significant.

###### 3.6.1.3.2 Response times

**Figure 2:**
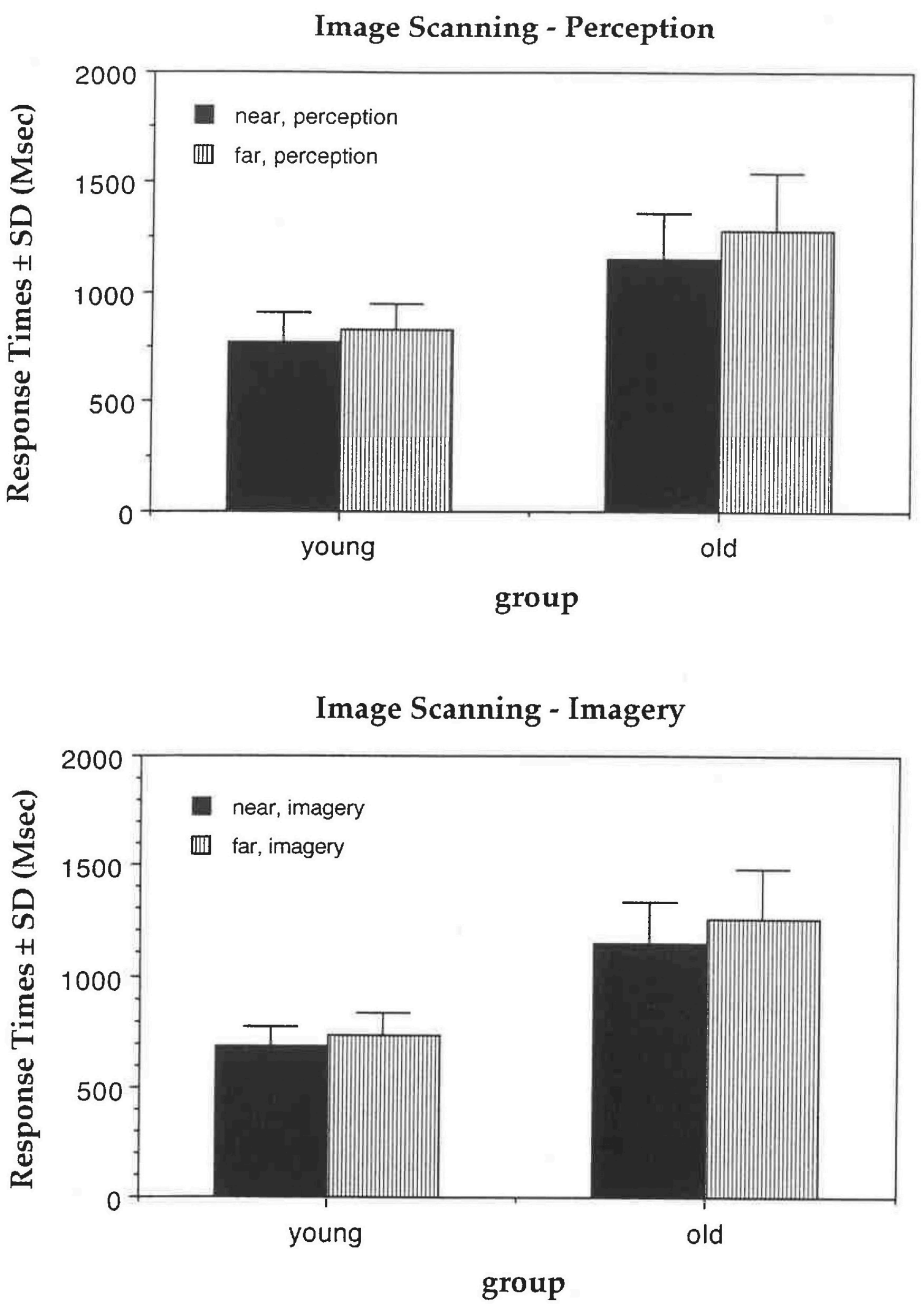
Image scanning task. In the perception condition, old participants were disproportionately slowed with the far arrows compared to the near arrows relative to the young. In the imagery condition, old participants showed a trend for slower response times with the far arrows relative to the near arrows compared to the young.

In the perception condition, old participants were overall slower than young participants, *F*_(1, 62)_ = 91.08, *p* = 0.0001 (means of 1,219 and 797 ms). All participants were slower with far than near arrows, *F*_(1, 62)_ = 35.29, *p* = 0.0001 (means of 1056 and 959 ms). Old participants were disproportionately slowed by far relative to near arrows compared to young participants, *F*_(1, 62)_ = 5.44, *p* = 0.02 (Figure 2), suggesting an impact of age-related changes in image scanning.

In the imagery condition, old participants were overall slower than young participants, *F*_(1, 62)_ = 161.59, *p* = 0.0001 (with means of 1,196 and 714 ms). All participants were slower with far relative to near arrows, *F*_(1, 62)_ = 20.88, *p* = 0.0001 (with means of 994 and 915 ms).

Old participants also showed a trend toward slower response times with far relative to near arrows compared to young participants, *F*_(1, 62)_ = 3.31, *p* = 0.07 (Figure 2), suggesting a trend for age-related changes in image scanning.

In summary, response times were paradoxically generally faster in the imagery condition than in the perception condition, possibly because participants in the perception condition double-checked their responses before pressing a response key. Our results indicate specific age-related changes in image scanning. However, our findings contrast with those of Dror and Kosslyn (1994), who did not identify a significant age interaction in the imagery version of this task. Our results suggest that elderly participants are less efficient than the young when scanning visual images.

### 3.7 Image maintenance

Image maintenance refers to the ability to hold images in memory for a few seconds. This task was originally developed by Kosslyn et al. (1990). Dror and Kosslyn (1994) evaluated age differences in the imagery condition of this task and found that old participants were overall slower and made more errors than young participants. In this task, participants studied the location of either two or four gray squares presented within an imaginary 4 × 5 grid delimited at the corners by four brackets, and then pressed a spacebar. In the perception condition, the gray squares were visible when an “X” appeared, at which point the participants decided whether the “X” covered one of either two or four squares. In the imagery condition, the gray squares disappeared when the spacebar was pressed, and when the “X” appeared participants decided whether it would have covered one of the gray squares.

#### 3.7.1 Method

##### 3.7.1.1 Materials

Four brackets were presented centrally on the computer screen forming the corners of an imaginary 4 × 5 grid. Either two (easy condition) or four (difficult condition) gray squares were presented within the brackets. Three seconds later, an “X” appeared somewhere within the brackets. Stimuli subtended a visual angle of 4° both horizontally and vertically. Number of squares (2, 4), location of the “X” (right or left, up or down), and location of the target square (left, right, up, or down) were all counterbalanced across the task.

##### 3.7.1.2 Procedure

An exclamation point first appeared at the center of the screen. When ready, participants pressed the spacebar to begin the next trial. After 500 ms, a set of brackets appeared in the center of the screen. Either two or four gray squares appeared inside the brackets. Participants studied the location of the gray squares and then pressed the spacebar. In the perception condition, a blank screen was presented for 50 ms, followed by the set of squares displayed for 2,950 ms. During this time, an “X” appeared within the brackets, and participants decided whether the “X” covered one of the gray squares.

In the imagery condition, after studying the location of the gray squares, participants pressed the spacebar, and the pattern disappeared. After 3 s, the set of brackets returned to the screen, with an “X” inside the brackets; this time the gray squares were no longer present, and participants decided whether the “X” fell in a location previously occupied by a gray square.

##### 3.7.1.3 Results

###### 3.7.1.3.1 Error rates

In the perception condition, no main effects or interactions were significant.

In the imagery condition, old participants made more errors overall than the young participants, *F*_(1, 62)_ = 14.19, *p* = 0.0004 (with means of 13% and 5%). Moreover, there was a trend for all participants to make more errors with four squares than with two squares, *F*_(1, 62)_ = 3.21, *p* = 0.08 (with means of 11% and 7%). However, the age × complexity interaction was not significant, *F* < 1.

###### 3.7.1.3.2 Response times

**Figure 3:**
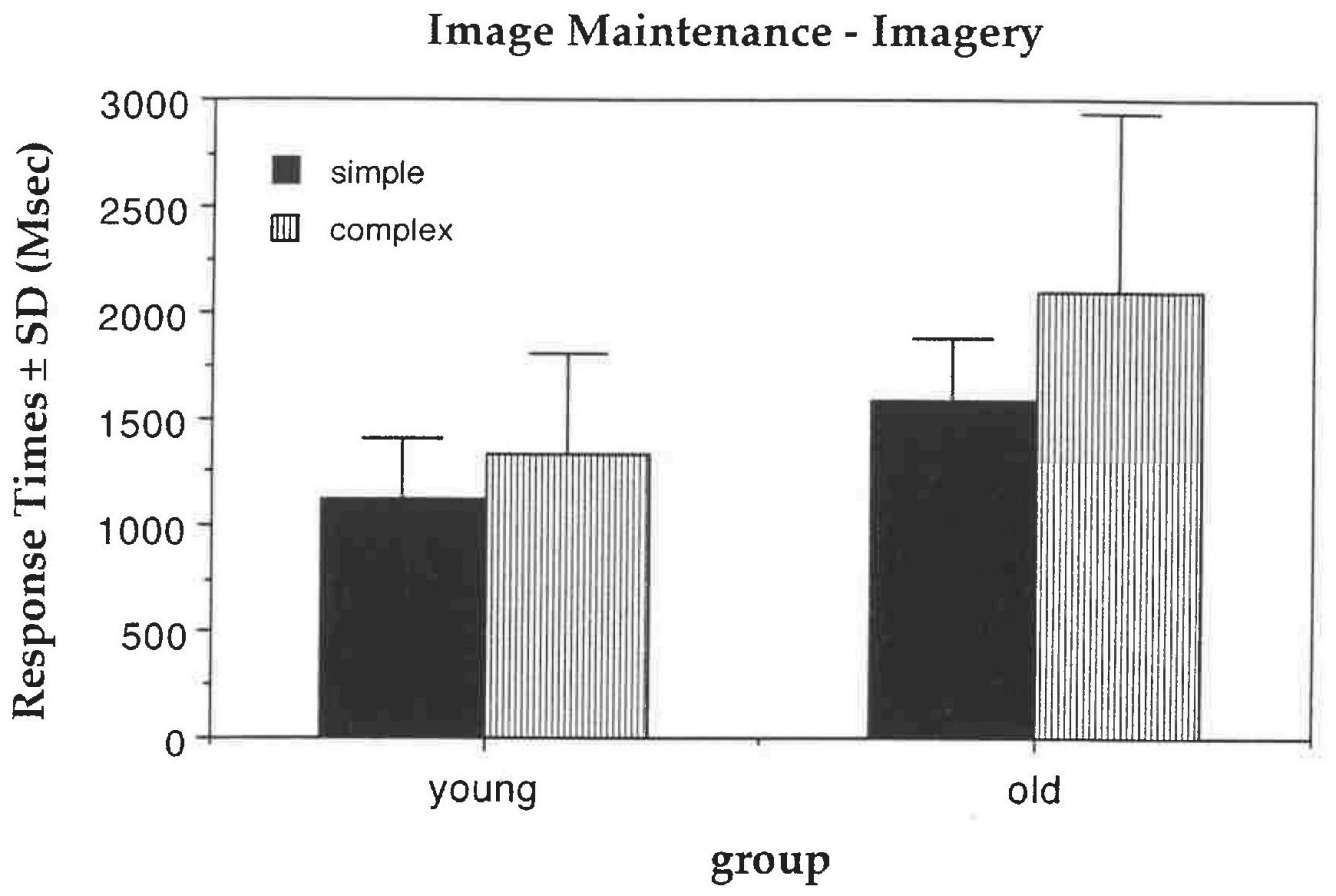
Image maintenance task. In imagery, old participants were disproportionately slowed relative to the young with four squares compared to two.

In the perception condition, old participants were slower than the young participants, *F*_(1, 62)_ = 38.13, *p* = 0.0001 (with means of 1,120 ms and 720 ms). Old participants were disproportionately slowed relative to the young participants when there were four squares compared to two squares, *F*_(1, 62)_ = 5.50, *p* = 0.02 (Figure 3). No other main effects or interactions were significant.

In the imagery condition, old participants were slower than the young, *F*_(1, 62)_ = 31.90, *p* = 0.0001 (with response times of 1,849 and 1,221 ms). Old participants were disproportionately slowed relative to the young when there were four squares compared to two, *F*_(1, 62)_ = 5.50, *p* = 0.02 (Figure 3). As expected, in the imagery condition, all participants were slower with four squares compared to two, *F*_(1, 62)_ = 28.13, *p* = 0.0001 (with means of 1,717 and 1,353 ms).

The results obtained in the imagery condition of this task for response times differ from those of Dror and Kosslyn (1994). In that study, the interaction between age and complexity was not significant for either response times or error rates. In our study, however, old participants had disproportionately longer response times with a greater number of squares in the display, suggesting that they took more time than the young to maintain a greater number of squares in memory and to decide whether an “X” had been on one of them. These results suggest that there are age-related changes in image maintenance in old compared to young participants, and that although performance can be maintained, this is at the cost of slowed response times.

### 3.8 Spatial imagery

In the perception condition, participants saw a 2 × 2 grid with a ball at the center. They then heard a set of directions while simultaneously observing the ball moving in each specified direction. In the imagery condition, participants heard a set of directions and imagined the ball traveling in the stated directions on an imaginary grid. When they heard a cue (the word “above,” “below,” “right,” or “left”), participants decided whether it accurately described the ball's final location relative to its initial position.

#### 3.8.1 Method

##### 3.8.1.1 Materials

In the perception condition, a 2 × 2 inch grid was presented at the center of the computer monitor, with a ball at its center. Each trial consisted of a series of directions (using the terms east, west, north, south, southeast, southwest, northeast, northwest) spoken by the computer, followed by a spoken cue (right, left, above, or below). In the perception condition, the ball moved in the stated direction within the square grid. In the imagery condition, neither the grid nor the ball was visible. Stimuli subtended a visual angle of 5.7° both horizontally and vertically. The number of directions (4, 5, 6, and 7) varied across trials. Short directions included 4 or 5 directions, while long directions included 6 or 7 directions. This variation was implemented to prevent participants from disregarding the first few trials and to ensure they paid attention throughout the experiment. The number of directions and the directions in which the ball moved were counterbalanced across trials.

##### 3.8.1.2 Procedure

In the perception condition, the participant heard a set of directions and simultaneously saw a ball moving from one location to another in the corresponding direction along a square grid. After the final direction, the participant heard a cue and decided whether the word accurately described the ball's final location relative to its initial location on the grid. Each trial started with a fixation point. After 500 ms, participants heard the first direction and saw the ball moving at a constant speed in that direction. When the ball finished moving on the grid, participants pressed the spacebar to hear a new direction and see the ball move to it. After the last direction was spoken and the ball moved to the final location, the participant heard one of the four cues and had to decide whether the cue accurately described the ball's final location relative to its initial location on the grid. If the cue accurately described this relationship, participants pressed the “Y” key; if not, they pressed the “N” key.

In the imagery condition, participants heard the set of directions but were asked to visualize the ball moving in the stated directions along an imaginary grid. After the final direction, participants heard a cue and decided whether it accurately described the ball's final location relative to its initial location on the grid.

##### 3.8.1.3 Results

###### 3.8.1.3.1 Error rates

In the perception condition, old participants made an overall equivalent number of errors as the young, *F* < l (with means of 6% and 4%, respectively). For all participants, the number of directions (short vs. long) had no effect on error rates (F < 1). Old participants did not make disproportionately more errors with the long directions than with the short directions in perception, *F*_(1, 62)_ = 1.27, *p* = 0.26 (with means of 5% and 6% for the old group, and 5% and 3% for the young group, for short and long directions, respectively).

In the imagery condition, old participants made an overall equivalent number of errors as the young (with means of 13% vs. 8%, respectively), *F*_(1, 62)_ = 2.54, *p* = 0.12. For all participants, the number of directions (short vs. long) had no effect on error rates (*F* < 1). Old participants did not make disproportionately more errors with the long directions than with the short directions in imagery, *F* < 1 (with means of 13% and 12% in old participants, and of 8% and 8% in young participants for short and long directions, respectively).

###### 3.8.1.3.2 Response times

In the perception condition, older participants were slower than the young, *F*_(1, 62)_ = 48.73, *p* = 0.0001 (with means of 1,931 and 1,189 ms). All participants were faster with the long directions than with the short directions (with means of 1,490 and 1,630 ms, respectively), *F*_(1, 62)_ = 19.76, *p* = 0.0001. Old participants tended to be disproportionately slower than the young with the short directions relative to the long directions (with means of 2,032 and 1,831 ms for old participants vs. 1,229 ms and 1,150 ms for young participants for short and long directions, respectively), *F*_(1, 62)_ = 3.80, *p* = 0.056.

In the imagery condition, old participants were slower than the young, *F*_(1, 62)_ = 39.71, *p* = 0.0001 (with means of 2,095 and 1,319 ms). All participants were marginally faster with the long directions than with the short directions (with means of 1,651 and 1,763 ms for long and short directions, respectively), *F*_(1, 62)_ = 3.86, *p* = 0.054. In imagery, the age × directions interaction was not significant, *F* < l.

Thus, contrary to expectations, these results indicate that all participants took longer with shorter directions than with longer directions, and that old participants tended to be disproportionately slowed relative to the young with the shorter directions compared to the longer directions in perception. The participants, who did not know when a trial was going to end, may have been better prepared to respond after a long set of directions because the end of the trial would then become more likely.

### 3.9 Imagery for objects

Variations in the response times and error rates of the following four tasks should primarily reflect the efficacy of processing in the ventral system. The first task is a modification of one developed by Eddie and Glass (1981). We used high-imagery sentences similar to those described by Eddie and Glass (1981). This task involved listening to a sentence that described a common property of an object or animal while the object was either pictured on the screen or mentally imagined. Participants decided whether the sentence was true or false.

#### 3.9.1 Methods

##### 3.9.1.1 Materials

In the perception condition, participants were shown 16 line drawings of objects or animals one at a time. Simultaneously, they heard a sentence describing a feature or property of the object or animal. In the imagery condition, participants heard a sentence describing a property of the object or animal; similar statements were validated by Eddie and Glass (1981) as requiring imagery. In this condition, participants had to visualize the objects or animals to determine whether the sentence was true or false. Line drawings were selected from the Snodgrass picture set (Snodgrass and Vanderwart, 1980). All pictures subtended a maximum visual angle of 7° both vertically and horizontally.

##### 3.9.1.2 Procedure

In the perception condition, an exclamation point appeared at the center of the screen. After 500 ms, a line drawing was presented along with a sentence describing a particular feature of the object or animal. In the imagery condition, only the sentence was heard; no picture was displayed. Participants decided, based either on the picture presented on the computer screen or on their mental image, whether the sentence was true or false. If the sentence was true, participants pressed the “Y” key; otherwise, they pressed the “N” key.

##### 3.9.1.3 Results

###### 3.9.1.3.1 Error rates

The two groups of participants made equivalent numbers of errors in both the perception and imagery conditions, both *F* < 1.

###### 3.9.1.3.2 Response times

Old participants were slower than young participants in the perception condition, *F*_(1, 62)_ = 25.83, *p* = 0.0001 (with means of 3,038 and 2,669 ms), and in the imagery condition, *F*_(1, 62)_ = 13.01, *p* = 0.0006 (with means of 3,176 and 2,874 ms).

### 3.10 Face imagery

In this task, participants determined which of two famous faces appeared rounder.

#### 3.10.1 Method

##### 3.10.1.1 Materials

In the perception condition, participants compared the faces of two famous people. Black-and-white photographs of these individuals were taken from biographies, magazines, and other books, featuring politicians, actors, and singers (such *as* Kevin Costner, Bill Clinton, and Charlie Chaplin). All photographs depicted the famous faces from the front, facing slightly to the right or left, with all facial features visible. Faces subtended a visual angle of 7 degrees horizontally and 7 degrees vertically and were presented to the right and left of the midline. Simultaneously, participants heard the names of these famous people spoken aloud by the computer speaker. In the imagery condition, participants only heard the names of pairs of famous people and decided whether the first person had a rounder face than the second based on their mental images of the faces.

In both conditions, each pair of faces was repeated twice, in the opposite order, due to the limited number of famous faces recognized by a large proportion of participants. A repeated pair of faces was not presented until all faces had been shown once. Half the photographs were of females, and the other half were of males. Half the time, two same-sex faces were compared, and half the time, a female face was compared to a male one.

##### 3.10.1.2 Procedure

In the perception condition, a fixation point was presented until participants pressed the spacebar. After 500 ms, two famous faces were displayed side by side, one appearing 500 ms after the other. At the same time, the names of the famous faces were spoken by the computer. In the imagery condition, participants only heard the names of two famous people 500 ms apart and had to visualize the faces. Participants then determined whether the first face was rounder than the second, either based on the photographs presented on the computer screen or on their own mental images of the faces. If the first face was rounder than the second, participants pressed the “Y” key; if it was not, they pressed the “N” key. The next pair was presented after a response was made.

##### 3.10.1.3 Results

###### 3.10.1.3.1 Error rates

Old participants made a similar number of errors as the young participants in the perception condition, *F* < 1, and in the imagery condition, *F*_(1, 62)_ = 2.15, *p* = 0.15.

###### 3.10.1.3.2 Response times

Old participants were slower than the young participants in the perception condition, *F*_(1, 62)_ = 14.75, *p* = 0.0003 (with means of 1,895 and 1,315 ms), and in the imagery condition, *F*_(1, 62)_ = 8.39, *p* = 0.005 (with means of 2,477 and 2,022 ms).

### 3.11 Color imagery

In this task, participants evaluated the truth of statements about the colors of specific objects, either based on a picture or on a mental image.

#### 3.11.1 Method

##### 3.11.1.1 Materials

Stimuli were close-up photographs of typical food items in one of four colors: red (4 trials), green (4 trials), yellow (4 trials), or brown (4 trials). Items were photographed against a gray background, then scanned into the computer and cropped. If needed, color balance was adjusted. The photographs subtended a maximum visual angle of 7° both horizontally and vertically. Sentences were spoken by the computer speaker.

##### 3.11.1.2 Procedure

A central fixation point was presented until the participant pressed the spacebar. In the perception condition, after 500 ms, a picture of a food item was displayed at the center of the computer screen, accompanied by a sentence describing the object's color. The sentence spoken by the computer included a qualifier such as “light” and “dark” (version 1) or “pale” and “deep” (version 2) (for example: “parsley is dark green” or “the inside of a banana is deep yellow”). Qualifiers were balanced across participants and conditions. In the imagery condition, participants heard only the sentence describing the color of the food item—no photograph was presented—and were asked to visualize the object. Participants decided, either based on the photograph displayed on the computer screen or their own mental image, whether the sentence was true or false. If the sentence was true, participants pressed the “Y” key; if it was false, they pressed the “N” key. The next stimulus was presented after a response was entered.

##### 3.11.1.3 Results

###### 3.11.1.3.1 Error rates

Old participants made more errors than the young in the perception condition, *F*_(1, 62)_ = 5.46, *p* = 0.02 (with means of 15% and 9%). In the imagery condition, their error rates were comparable, *F* < 1.

###### 3.11.1.3.2 Response times

Old participants were slower than the young in the perception condition, *F*_(1, 62)_ = 14.77, *p* = 0.0003 (with means of 2,661 and 2,191 ms), and in the imagery condition, *F*_(1, 62)_ = 9.56, *p* = 0.003 (with means of 3,026 and 2,572 ms).

It is interesting to note that age-related changes in color discrimination have been reported (e.g., Haegerstrom et al., 1999). Our results suggest that these age-related changes do not affect imagery for colors—even though they may affect judgments based on the perception of colors.

### 3.12 Auditory imagery

Participants decided whether the first or the second of two successively presented sounds (or names of objects in the imagery condition) was higher in pitch than the second. This task differs from the others in that it relies on hearing rather than vision. Given that elderly participants show age-related changes in hearing (e.g., Schieber, 1992), we thought it would be interesting to determine whether these changes could affect imagery of sounds.

#### 3.12.1 Method

##### 3.12.1.1 Materials

Participants decided which of two sounds was higher in pitch. Sounds were 2 s long and were obtained from commercial CDs. They included common sounds of animals and objects. Sounds were presented one after another with an inter-stimulus interval of 500 ms. If the first sound was higher in pitch, participants pressed the “Y” key; if it was not, they pressed the “N” key.

##### 3.12.1.2 Procedure

In the perception condition, an exclamation point was presented. After participants pressed the spacebar, two sounds were presented with a 500 ms interval in between. Participants decided whether the first sound was higher in pitch than the second based on what they heard. After a response was entered, the exclamation point returned to the computer screen.

In the imagery condition, participants heard the names of two animals or objects spoken by the computer speaker 500 ms apart and had to imagine whether the sound emitted by the first object was higher in pitch than that emitted by the second object.

##### 3.12.1.3 Results

###### 3.12.1.3.1 Error rates

Old participants made more errors than the young in the perception condition, *F*_(1, 62)_ = 20.95, *p* = 0.0001 (with means of 26% and 13%), and in the imagery condition, *F*_(1, 62)_ = 5.57, *p* = 0.02 (with means of 30% and 24%).

###### 3.12.1.3.2 Response times

Old participants tended to be slower than the young in both perception, *F*_(1, 62)_ = 3.26, *p* = 0.08 (with means of 2,434 and 2,121 ms), and imagery, *F*_(1, 62)_ = 3.49, *p* = 0.07 (with means of 3,152 and 2,693 ms).

These results are consistent with the presence of age-related changes in the perception and imagery of sounds.

### 3.13 Word imagery

The following three tasks were classified as not relying predominantly on ventral or dorsal processing, but rather as relying on mixed dorsal/ventral processing. Ventral processing is used to retrieve the objects to be compared from long-term memory, while dorsal processing is involved in making the judgments. The word imagery task is a modification of a task originally designed by Weber and Castelman (1970) and Weber and Malmstrom (1979).

#### 3.13.3.1 Method

##### 3.13.1.1 Materials

Twenty-four low-imagery, high-frequency (AA, Thorndike and Lorge, 1966) four-letter verbs were chosen (e.g., “live” and “wish”). The first and last letters of these words were either both tall (as in “hold,” 4 trials), both short (as in “move,” 4 trials), the first tall and the last short (as in “live,” 4 trials), or the first short and the last tall (as in “meet,” 4 trials). In the perception condition, verbs were presented centrally on the computer screen in a black 24-point Chicago plain font on a white background and subtended a visual angle of 3.4° horizontally and 1.2° vertically. In the imagery condition, verbs were presented auditorily through the computer speaker, and participants visualized them printed in lowercase letters.

##### 3.13.1.2 Procedure

A central fixation point was displayed. After 500 ms, a stimulus was presented. In the perception condition, a lowercase letter word appeared at the center of the computer screen. The word remained on the screen until a response key was pressed. In the imagery condition, the word was spoken by the computer. In both conditions, participants decided whether the first and last letters of the words were the same height. If they were, participants pressed the “Y” key; if they were not, participants pressed the “N” key. An exclamation point then returned to the screen to signal the beginning of a new trial.

##### 3.13.1.3 Results

###### 3.13.1.3.1 Error rates

The two groups of participants made equivalent numbers of errors in the perception condition, *F* < 1, and in the imagery condition, *F*_(1, 62)_ = 1.70, *p* = 0.20.

###### 3.13.1.3.2 Response times

Old participants were slower than the young in both perception, *F*_(1, 62)_ = 61.59, *p* = 0.0001 (with means of 1,403 and 941 ms), and imagery, *F*_(1, 62)_ = 41.17, *p* = 0.0001 (with means of 2,341 and 1,758 ms).

### 3.14 Size imagery

This task involves judging the relative size of a pair of objects. Although the task requires comparing pairs of objects based on information retrieved from long-term memory, the judgment is spatial.

#### 3.14.1 Method

##### 3.14.1.1 Materials

Participants compared two items, which were either both animals (4 trials), both objects (4 trials), or one animal and one object (8 trials). Stimuli were line drawings of objects and animals from the Snodgrass and Vanderwart (1980) set. Stimuli subtended a maximum visual angle of 7° to the right or left of a central fixation point. In both perception and imagery, participants heard the names of the objects, which were presented by the computer.

##### 3.14.1.2 Procedure

A central fixation point was displayed until participants pressed the spacebar. After 500 ms, a stimulus was presented. In the perception condition, the stimulus was a line drawing of an object or animal presented to the left of the central fixation point on the computer monitor. At the same time, participants heard the name of the item spoken by the computer. After 500 ms, a second item was presented to the right side of the central fixation point, and its name was spoken. In the imagery condition, participants heard the name of the first object or animal, and after 500 ms, they heard the name of the second object or animal. Participants were asked to visualize the objects or animals in their most standard position. In both conditions, participants decided whether the first object was taller than the second at its highest point. If the first object was taller than the second, participants pressed the “Y” key; if it was not, they pressed the “N” key.

##### 3.14.1.3 Results

###### 3.14.1.3.1 Error rates

The two groups of participants made equivalent numbers of errors in the perception condition, *F*_(1, 62)_ = 2.00, *p* = 0.16, and in the imagery condition, *F* < 1.

###### 3.14.1.3.2 Response times

Old participants were slower than the young in the perception condition, *F*_(1, 62)_ = 25.55, *p* = 0.0001 (with means of 1,541 and 1,028 ms), and in the imagery condition, *F*_(1, 62)_ = 11.40, *p* = 0.001 (with means of 3,118 and 2,322 ms).

### 3.15 Tactile imagery

In this task, participants evaluated the firmness of pairs of objects. They heard the names of two common objects or animals and decided whether the first object was firmer than the second object. This task was classified as mixed because, although participants had to retrieve images of objects from long-term memory, tasks involving similar types of judgments activated the sensory integration regions in the parietal association areas (Roland and Mortensen, 1987; Uhl et al., 1994).

#### 3.15.1 Method

##### 3.15.1.1 Materials

Participants heard the names of two objects and decided whether the first was firmer than the second. The task included 48 trials and was based on 24 pairs of stimuli presented twice each. Half the trials involved far discriminations (such as “bear-rock”) and half involved close discriminations (such as “banana-tube of toothpaste”). The far trials corresponded to the control condition (which is not likely to involve imagery), whereas the close trials corresponded to the imagery condition (which probably involves the use of imagery; see Kosslyn et al., 1977 for a justification of these assumptions; see also Berardi, 2025). All pairs of items were spoken by the computer in a male voice. After the first presentation of the 24 pairs, all pairs were presented again in a different order. The item that was presented first in the first occurrence was now presented second and vice versa. Participants decided whether the first item was firmer to the touch than the second. If the first object was firmer, participants pressed the “Y” key; if it was not, they pressed the “N” key. There were equal numbers of “yes” and “no” responses.

##### 3.15.1.2 Procedure

Participants saw an exclamation point at the center of the computer screen. After 500 ms, a word was spoken by the computer speaker, followed after another 500 ms by a second word. Participants decided whether the first named object was firmer than the second. After a response key was pressed, the exclamation point returned to the screen to signal the beginning of a new trial.

##### 3.15.1.3 Results

###### 3.15.1.3.1 Error rates

Old and young participants made equivalent numbers of errors in the control condition, *F* < 1, and in the imagery condition, *F* < 1.

###### 3.15.1.3.2 Response times

Old participants were slower than the young in both control, *F*_(1, 62)_ = 15.72, *p* = 0.0002 (with means of 2,008 and 1,583 ms), and imagery conditions, *F*_(1, 62)_ = 23.75, *p* = 0.0001 (with means of 3,145 and 2,126 ms).

### 3.16 Comparing tasks

The results show that for all tasks, whether dorsal, ventral, or mixed, old participants were generally slower than the young in processing information in both perceptual and imagery conditions. For the spatial tasks, old participants had equivalent error rates to the young in the spatial and motor imagery tasks in both perceptual and imagery conditions. However, old participants made more errors than the young in the imagery condition of the image rotation, image maintenance, image scanning, and brackets image generation tasks. They also showed a trend toward making more errors in the imagery condition of the grids image generation task. The error rates in the perceptual conditions of these tasks were equivalent to those of young participants, except for the brackets image generation task, in which old participants tended to make more errors than the young. Error rates of old participants were equivalent to those of the young for the three mixed tasks, namely word, tactile, and size imagery, in both perceptual and imagery conditions. The accuracy of young and elderly participants was comparable for two ventral tasks (face and object imagery) in both perceptual and imagery conditions. However, in the color imagery task, old participants made more errors than the young in perception (in imagery, the error rates of the two groups were equivalent). In the auditory imagery task, old participants made more errors than the young in both perceptual and imagery conditions.

To determine whether old participants showed greater decrements in one type of task (dorsal, ventral, or mixed), we carried out an analysis of variance with one between-group factor (age) and one within-participants factor (type of task). For this analysis, we used mean response times and mean error rates for all ventral tasks (*n* =4), all dorsal tasks (*n* =7), and all mixed tasks (*n* = 3). We obtained the following results:

#### 3.16.1 Perception error rates

Old participants made more errors overall than the young (with means of 8% and 6% respectively), *F*_(1, 62)_ = 9.96, *p* = 0.003. All participants made more errors on ventral tasks than on dorsal tasks and more errors on mixed tasks than on dorsal tasks (with means of 12%, 6%, and 2% for ventral, mixed, and dorsal tasks, respectively), *F*_(2, 124)_ = 82.56, *p* = 0.0001. All orthogonal contrasts were significant (ventral vs. dorsal, *F* = 156.52, *p* = 0.0001; dorsal vs. mixed, *F* = 13.80, *p* = 0.0003; ventral vs. mixed, *F* = 77.37, *p* = 0.0001). Old participants made disproportionately more errors than the young in ventral tasks compared to dorsal or mixed tasks (with means of 10%, 4%, and 3% for young and 15%, 7%, and 3% for old, in ventral, mixed, and dorsal tasks, respectively).

#### 3.16.2 Perception response times

Old participants were overall slower than the young (with means of 1,848 and 1,336 ms respectively), *F*_(1, 62)_ = 57.90, *p* = 0.0001. The main effect of type of task was significant (with means of 2,291, 1,228, and 1,258 ms for ventral, mixed, and dorsal tasks, respectively). Orthogonal contrasts indicated that all participants were slower with the ventral than with the mixed (*F* = 669.28, *p* = 0.0001) or dorsal tasks (*F* = 633.01, *p* = 0.0001); however, the mixed and dorsal tasks did not differ (*F* < 1). The interaction of age and type of task was only marginally significant (with means of 2,074, 985, and 950 ms in young and 2,507, 1,472, and 1,565 ms in old for ventral, mixed, and dorsal tasks respectively), *F*_(2, 124)_ = 2.56, *p* = 0.08.

#### 3.16.3 Imagery error rates

Old participants made overall more errors than the young (with means of 18% and of 15%, respectively), *F*_(1, 62)_ = 7.29, *p* = 0.009. The main effect of type of task was significant, *F*_(2, 124)_ = 50.25, *p* = 0.0001. Orthogonal contrasts indicated that all participants made more errors with ventral than with mixed (*F* = 36.22, *p* = 0.0001) or dorsal tasks (*F* = 99.05, *p* = 0.0001). They also made more errors with mixed than dorsal tasks (*F* = 15.47, *p* = 0.0001). The interaction of age and type of task, however, was not significant, *F*_(2, 124)_ = 1.24, *p* = 0.29.

#### 3.16.4 Imagery response times

Old participants were overall slower than the young (with means of 2,720 and 2,023 ms, respectively), *F*_(1, 62)_ = 42.59, *p* = 0.0001. The main type of task effect was significant (with means of 2,749, 2,385, and 1,981 ms for ventral, mixed, and dorsal tasks, respectively), *F*_(2, 124)_ = 12.36, *p* = 0.0001. Orthogonal contrasts indicated that all types of tasks differed from each other (ventral from mixed, *F* = 40.97, *p* = 0.0001; ventral from dorsal, *F* = 182.28, *p* = 0.0001; and mixed from dorsal, *F* = 50.42, *p* = 0.0001). The interaction of age and type of task was significant (with means of 2,540, 2,040, and 1,490 ms for young participants and 2,957, 2,729, and 2,472 ms for old participants in ventral, mixed, and dorsal tasks, respectively), *F*_(2, 124)_ = 12.36, *p* = 0.0001.

We then considered the number of tasks on which old participants showed age-related changes relative to the young. For response times, old participants were consistently slower than the young in both perception and imagery conditions. For error rates in the perception condition, old participants showed age-related changes in 2 out of 4 ventral tasks and in 1 out of 7 dorsal tasks. In the imagery condition, old participants showed age-related changes in 1 out of 4 ventral tasks and in 5 out of 7 dorsal tasks.

The results of these analyses indicate that all participants were slower and made more errors with ventral tasks than with dorsal tasks. Old participants were also disproportionately slower relative to the young in ventral tasks compared to dorsal tasks. However, the number of tasks actually showing age-related changes is greater for dorsal than for ventral tasks in imagery.

These results are particularly interesting because all IPB tasks were self-paced. Our findings indicate that even when given as much time as needed, old participants exhibit age-related changes in perception and mental imagery tasks. It is possible that the slower response times may be explained by the generalized slowing hypothesis (Cerella, 1985, 1990; Salthouse, 1985, 1996, 2000). However, when we compared logarithmically transformed response times in young and elderly participants, significant differences remained for all tasks. In the imagery conditions, logarithmically transformed values for old participants differed from those of the young for the tactile, image rotation, word, motor, maintenance, image scanning, spatial, grids, and brackets tasks (all *p* = 0.0001). They also differed in imagery for the size (*p* = 0.0002), object (*p* = 0.0006), color (*p* = 0.001), face (*p* = 0.002), and auditory (*p* = 0.04) tasks. In perception, they differed on the face, object, spatial, image rotation, word, motor, size, image maintenance, scanning, grids, and brackets tasks (all *p* = 0.0001). They also differed for the tactile and color tasks (*p* = 0.0002), but only marginally for the auditory task (*p* = 0.09). These results indicate that the slowness of the elderly relative to the young participants is greater than would be predicted by the simplest version of the generalized slowing hypothesis.

### 3.17 Nonmetric Multi-dimensional scaling analyses

Nonmetric multi-dimensional scaling (MDS) was used to determine whether in older participants the IPB tasks would form less clearly delimited clusters than those found in young participants. The correlation matrices for perception and imagery tasks in old participants are shown in Supplementary Tables 1a, b. The corresponding matrices for young participants are shown in Supplementary Tables 1c, d. The obtained correlation matrices were analyzed with MDS based on monotonicity coefficients (Shepard, 1962; Kruskal, 1964) to determine the spatial distribution of 14 of the 15 IPB tasks. The response training task was designed to familiarize the participants with the procedures to be used for all tasks, and therefore these data were not included in the following analyses. The same analyses were conducted separately for the imagery data. Response accuracy was not analyzed further because of near error-free performance in some tasks and close to zero correlations between most tasks (see Tables 1a, b, and Supplementary Tables 1b, d). MDS leads to spatial representations of relationships among variables in which “distances” among variables are represented as Euclidean distances. Factor analysis was used to obtain independent confirmation of the MDS results. Multiple regression analyses were carried out to determine which task attributes best correlated with the MDS dimensions.

When not mentioned in the text, means and standard deviations for young and old participants' performance for all tasks and conditions are presented in Tables 1a, b.

Results that are not mentioned were not significant.

#### 3.17.1 Perceptual analyses

**Figure 4:**
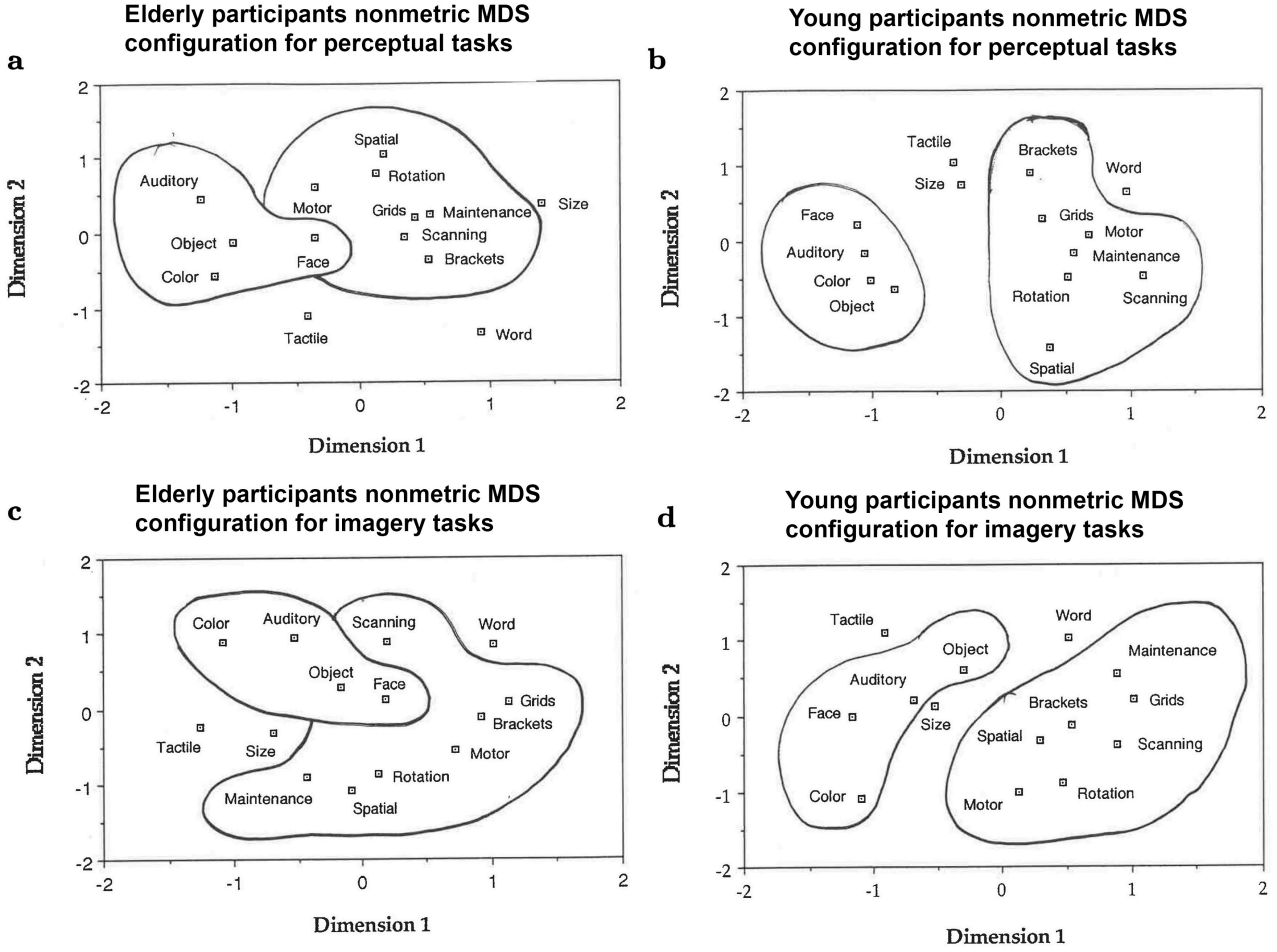
**(a–d)** Nonmetric MDS configurations for perceptual and imagery tasks in old and young participants. In elderly participants, clusters of dorsal and ventral tasks are much less well-segregated than in young participants, particularly in the imagery condition. In young participants, clusters of dorsal and ventral tasks are well-segregated. Ventral tasks: object, auditory, face, and color imagery; dorsal tasks: image generation (brackets and grids), image scanning, maintenance, rotation, motor, and spatial imagery; mixed tasks: size, word, and tactile imagery. Figures 4b,d have been published originally in Berardi (2025).

The two-dimensional solution accounted for 85% of the variance and was deemed adequate (Figure 4a). The stress value was 0.16 after 16 iterations. This value is below the criterion for a 0.05 level of the distribution of stress for scaling 12 stimuli in a two-dimensional Euclidean space (stress value = 0.211). This criterion was slightly more stringent than necessary, given that we had 14 rather than 12 tasks. Our stress value is therefore significantly better than that for random data (see Klahr, 1969) and does not justify a higher-dimensional space. For the reader's information, the three-, four-, and five-dimensional solutions yielded stress values of 0.08, 0.05, and 0.03 and explained 95%, 97%, and 99% of the total variance, respectively.

Visual inspection of Figure 4a revealed two major clusters of tasks: the first cluster included image generation (grids and brackets), image maintenance, image rotation, motor imagery, spatial imagery, image scanning, tactile imagery, and face imagery. The second cluster included color, object, and auditory imagery tasks. The third cluster included word and size imagery. The first cluster mainly reflected dorsal processing tasks, the second ventral processing tasks, and the last mixed dorsal/ventral processing tasks. Interestingly, the face imagery task, which was classified as a ventral task, clustered closer to the spatial tasks. Moreover, comparing this graph with that obtained for young healthy participants (see Figures 4a, b), we can clearly see that the clusters are, although still reasonably well-defined, less distinctly separate for old than for young participants. Old participants show some overlap of dorsal/ventral clusters that we do not observe in young participants.

The second dimension, like that found for young participants, was interpreted as reflecting the degree of complexity of the stimuli.

#### 3.17.2 Imagery analyses

The two-dimensional solution was adequate and accounted for 77% of the variance, with a stress value of 0.18 after 18 iterations (see Figure 4c), compared to stress values of 0.10, 0.05, and 0.03 for 3, 4, and 5 dimensions, which explained 89%, 96%, and 98% of the variance, respectively. Again, the significance level for this stress value is *p* < 0.05 (Klahr, 1969), and this significance level (based on 12 stimuli) is slightly more stringent than required. Visual inspection of Figure 4c indicates one indistinct cluster containing all IPB tasks. Considering our classification into dorsal, ventral, and mixed processing tasks, we note that the overlap between the three clusters is now even more evident than in the perception condition. Size and word imagery, both mixed processing tasks we expected to be close to each other, are actually at opposite ends of the display.

After inspecting the solution, Dimension 2 was interpreted as the degree of complexity of the stimuli or perhaps the degree of memory load involved in processing the stimuli.

### 3.18 Factor analyses

Principal components analysis (PCA) was used to provide independent confirmation of the pattern of results obtained with MDS. Data were rotated using the varimax procedure. Because we posited that all tasks relied on dorsal, ventral, or a mixture of both types of processing, we sought to determine whether factor loadings would reflect these variables. Variables with factor loadings ≥0.40 were considered as loading on that factor.

#### 3.18.1 Perception analyses

In old participants, the first two factors explained 52% and 38% of the variance in the rotated factors for the perception condition, respectively. All eigenvalues were greater than 1, indicating the factors were important. Factor 1 had high loadings on spatial perception, image rotation, word imagery, motor imagery, image maintenance, image generation (grids and brackets), size imagery, and image scanning tasks. Surprisingly, the face imagery task also loaded significantly on this factor. Factor 1, therefore, represented mainly tasks mediated by the dorsal visual stream. Factor 2 loaded highly on face imagery, object imagery, color imagery, and auditory imagery tasks, as expected, but also on tactile imagery, image rotation and motor imagery tasks. Factor 2, therefore, loaded on all tasks mediated by the ventral visual stream and several tasks mediated by the dorsal visual stream. The size and word imagery tasks, which were expected to load highly on both factors, only loaded on factor 1, indicating that for elderly participants, the spatial component predominantly contributed to performance on these tasks. The tactile imagery task, which was also expected to load on both factors, had a somewhat moderate loading on the ventral factor, indicating that retrieval of objects from long-term memory was more important in this task (see Table 2a). The rotated factor loadings are shown in Table 2a.

**Table 2a:** Principal components analyses of the perceptual correlation matrix in old participants.

	**Component loadings**			
**IPB tasks**	**Unrotated**		**Rotated**	
	**l**	**2**	**1**	**2**
Face	0.820	0.134	**0.563**	**0.611**
Object	0.702	0.514	0.235	**0.837**
Spatial	0.740	−0.141	**0.669**	0.346
Tactile	0.616	0.317	0.289	**0.629**
Rotation	0.757	−0.068	**0.638**	**0.413**
Word	0.538	−0.186	**0.538**	0.186
Motor	0.794	0.114	**0.554**	**0.580**
Color	0.616	0.512	0.168	**0.783**
Maintenance	0.843	−0.388	**0.903**	0.215
Auditory	0.631	0.557	0.152	**0.828**
Grids	0.850	−0.319	**0.866**	0.274
Brackets	0.789	−0.223	**0.758**	0.311
Size	0.616	−0.439	**0.756**	0.035
Scanning	0.819	−0.149	**0.737**	0.389

Variables with factor loadings >0.40 are considered as loading on that factor and are in bold.

In young participants, the first two factors explained 44% and 32% of the variance in the rotated factors for the perception condition, respectively. Factor 1 had high loadings on spatial perception, image rotation, word imagery, motor imagery, image maintenance, image generation (grids and brackets), and image scanning tasks. Therefore, Factor 1 clearly represented tasks mediated by the dorsal system. Factor 2 showed high loadings on face imagery, object imagery, color imagery, and auditory imagery tasks. Factor 2, then, reflected predominantly tasks mediated by the ventral system. The size imagery and tactile imagery tasks had somewhat moderate loadings on both the ventral and dorsal factors, indicating that they involved, as expected, mixed dorsal/ventral processing (see Table 2c). Interestingly, the unrotated factor loadings showed that ventral tasks loaded negatively on the dorsal factor, and dorsal tasks loaded negatively on the ventral factor, indicating that the two factors are independent but correlated. The unrotated and rotated factor loadings are shown in Table 2c.

#### 3.18.2 Imagery analyses

In old participants, the first two factors explained 41% and 39% of the variance in the rotated factors, respectively. All eigenvalues were greater than 1, indicating that the factors were significant. Factor 1 loaded on spatial imagery, image rotation, motor imagery, word imagery, image generation (grids and brackets), and image scanning tasks, but also unexpectedly on face imagery and object imagery. Surprisingly, image maintenance did not load on this factor. Factor 1 loaded on most dorsal system tasks, but not all (e.g., the maintenance task). It also loaded on several ventral visual stream tasks, such as face imagery and object imagery. Factor 2 loaded on face imagery, object imagery, auditory imagery, and color imagery, representing ventral tasks. Tactile imagery and size imagery also loaded exclusively on Factor 2. Unexpectedly, image rotation and spatial imagery also loaded on Factor 2. The unrotated and rotated factor loadings are shown in Table 2b.

**Table 2b:** Principal components analyses of the imagery correlation matrix in old participants.

	**Component loadings**			
**IPB tasks**	**Unrotated**		**Rotated**	
	**1**	**2**	**1**	**2**
Face	0.763	−0.118	**0.631**	**0.445**
Object	0.741	0.105	**0.459**	**0.590**
Spatial	0.688	0.117	**0.414**	**0.562**
Tactile	0.597	0.613	0.003	**0.856**
Rotation	0.731	0.021	**0.511**	**0.523**
Word	0.572	−0.188	**0.542**	0.262
Motor	0.729	−0.386	**0.793**	0.229
Color	0.571	0.431	0.112	**0.707**
Maintenance	0.684	0.256	0.314	**0.659**
Auditory	0.662	0.130	0.386	**0.554**
Grids	0.662	−0.467	**0.801**	0.124
Brackets	0.701	−0.563	**0.896**	0.082
Size	0.716	0.338	0.280	**0.740**
Scanning	0.678	−0.155	**0.595**	0.360

Variables with factor loadings >0.40 are considered as loading on that factor and are in bold.

**Table 2c:** Principal components analyses of the perceptual correlation matrix in young participants.

	**Component loadings**			
**IPB tasks**	**Unrotated**		**Rotated**	
	**l**	**2**	**1**	**2**
Face	0.491	−0.695	0.004	**0.850**
Object	0.519	−0.530	0.152	**0.726**
Spatial	0.354	0.196	**0.404**	0.025
Tactile	0.557	−0.196	0.364	**0.465**
Rotation	0.631	0.124	**0.598**	0.235
Word	0.589	0.609	**0.824**	−0.196
Motor	0.719	0.360	**0.800**	0.084
Color	0.475	−0.655	0.048	**0.808**
Maintenance	0.777	0.257	**0.793**	0.201
Auditory	0.474	−0.573	0.091	**0.738**
Grids	0.851	0.207	**0.829**	0.283
Brackets	0.702	0.151	**0.673**	0.250
Size	0.731	−0.103	**0.561**	**0.480**
Scanning	0.533	0.255	**0.586**	0.072

Variables with factor loadings >0.40 are considered as loading on that factor and are in bold.

This table has been published originally in Berardi (2025).

In young participants, Factor 1 loaded on dorsal tasks: spatial imagery, image rotation, word imagery, motor imagery, image maintenance, image generation (grids and brackets), and image scanning tasks. Factor 2 loaded on ventral tasks, including face, object, tactile, color, auditory, and size imagery tasks. The tactile and size imagery tasks were more clearly associated with the ventral factor, perhaps because there is a greater reliance on long-term memory retrieval of the objects than in the perception condition. The unrotated and rotated factor loadings are shown in Table 2d.

**Table 2d:** Principal components analyses of the imagery correlation matrix in young participants.

	**Component loadings**			
**IPB tasks**	**Unrotated**		**Rotated**	
	**1**	**2**	**1**	**2**
Face	0.359	−0.599	0.005	**0.698**
Object	0.501	−0.342	0.257	**0.549**
Spatial	0.666	0.250	**0.701**	0.124
Tactile	0.285	−0.388	0.052	**0.481**
Rotation	0.606	0.147	**0.597**	0.182
Word	0.541	0.110	**0.522**	0.181
Motor	0.563	0.109	**0.541**	0.193
Color	0.168	−0.393	−0.055	**0.423**
Maintenance	0.635	0.362	**0.731**	0.012
Auditory	0.610	−0.652	0.194	**0.872**
Grids	0.617	0.495	**0.783**	−0.112
Brackets	0.780	0.269	**0.808**	0.166
Size	0.715	−0.545	0.338	**0.833**
Scanning	0.648	0.356	**0.739**	0.024

Variables with factor loadings >0.40 are considered as loading on that factor and are in bold.

This table has been published originally in Berardi (2025).

The dorsal/ventral distinction, then, accounted well for both the perception and imagery data in young participants. These results dovetail nicely with the results from the MDS analysis, even though the percentage of total variance accounted for by the factors was relatively low. In summary, we are confident that the first two factors reflect dorsal/ventral processing in both perception and imagery. In old participants, for both perception and imagery, the factors loaded on a greater number of tasks than they did in young participants, but the loadings are generally lower than those observed in young participants. In the young, we found that Factor 1 closely reflected dorsal stream processing, while Factor 2 reflected ventral stream processing. Mixed processing tasks had somewhat moderate loadings on both factors. In imagery, a similar pattern was found: Factor 1 reflected dorsal stream processing and Factor 2 reflected ventral stream processing. Two of the mixed tasks (size and tactile) were more closely associated with the ventral tasks in imagery, probably because images of objects had to be retrieved from long-term memory.

The results of the factor analyses support the findings of the MDS analyses. Both analyses also suggest that the dorsal and ventral visual pathways were much less well-segregated in healthy old participants than in young healthy participants in both visual perception and mental imagery. In mental imagery, several spatial tasks loaded on the ventral factor (e.g., spatial imagery, image maintenance, and image rotation), confirming the lesser segregation of the visual pathways behaviorally. The results also indicate that dorsal/ventral processing is nevertheless a strong component of both young and old participants' performance.

### 3.19 Multiple regression analyses

We also interpreted the MDS dimensions by correlating them with attributes that could theoretically reflect performance on the IPB tasks. Each task was rated on a scale from 1 to 7 for each attribute. The attributes considered were: the degree of dorsal/ventral processing required, the degree of auditory processing, the number of stimuli in each trial, the degree of concentration, memory load (in the imagery condition only), degree of verbal processing, the cerebral hemisphere predominantly involved, visual complexity, verbal complexity, the number of processes involved, and the degree of stimulus complexity (whether visual or verbal). Whenever these ratings changed across perceptual and imagery conditions, different ratings were used. Multiple regression analyses were then performed on the data from the perception and imagery tasks separately (Tables 3a–d). Two criteria had to be met before we inferred that an attribute best reflected an MDS dimension. First, the overall regression had to be statistically significant (*p* < 0.05); and second, the attribute with the highest standardized regression weight was taken to best reflect an MDS axis.

**Table 3a:** Elderly participants. Multiple r and multiple regression coefficients for predicting task attributes from MDS dimensions in perception.

**Attribute**	**Multiple R**	**Dimension 1**	**Dimension 2**
Auditory	0.54	−0.56	0.05
Dorsal/ventral	0.70^*^	0.69^*^	0.46
Verbal processing	0.41	−0.66	−0.45
Number of stimuli	0.08	−0.11	0.17
Concentration	0.34	−0.30	0.36
Number of processes	0.47	0.37	−0.32
Hemisphere	0.26	−0.11	0.15
Visual complexity	0.42	0.32	0.26
Verbal complexity	0.56	−0.35	−0.38
Stimulus complexity	0.71^*^	−0.32^*^	0.39^*^
Familiarity	0.59	−0.27	−0.71^*^

^*^p < 0.05, ^**^p < 0.01, ^***^p < 0.005.

In elderly participants, for the perception condition, Dimension 1 was significantly correlated with the dorsal/ventral ratings (*r* = 0.70, *p* < 0.05) and with stimulus complexity (*r* = 0.71, *p* < 0.05). Although these correlations were of comparable magnitude, the dorsal/ventral ratings were associated with a higher regression weight (β = 0.61) than stimulus complexity (β = −0.48). Dimension 2 correlated significantly with stimulus complexity (*r* = 0.71, *p* = 0.05), which was associated with the highest regression weight (β = 0.52). Stimulus familiarity also had a significant regression weight on Dimension 2, indicating that this attribute may have partially contributed to this dimension. However, because this variable explained only 35% of the variance and the overall multiple regression was not significant, stimulus complexity appears to be the best characterization of Dimension 2 (Table 3a).

Therefore, in the perception condition, Dimension 1 was taken to reflect dorsal/ventral processing, whereas Dimension 2 was taken to reflect stimulus complexity.

In elderly participants, for the imagery condition, the pattern of results was slightly different. Dimension 1 was primarily accounted for by the degree of concentration required by the tasks (Table 3b).

**Table 3b:** Elderly participants. Multiple r and multiple regression coefficients for predicting task attributes from MDS dimensions in imagery.

**Attribute**	**Multiple R**	**Dimension 1**	**Dimension 2**
Auditory	0.62	−0.67^*^	0.11
Dorsal/ventral	0.75^**^	0.56^*^	−0.74^**^
Verbal processing	0.48	−0.91	0.23
Number of stimuli	0.35	−0.20	−0.14
Concentration	0.75^**^	−0.95^***^	−0.31
Memory load	0.69	0.08	−1.04^**^
Number of processes	0.008	0.02	0.10
Hemisphere	0.49	−0.30	−0.15
Visual complexity	0.20	0.19	−0.02
Verbal complexity	0.52	−0.25	0.41
Stimulus complexity	0.50	−0.42	−0.25
Familiarity	0.39	−0.15	0.47

^*^p < 0.05, ^**^p < 0.01, ^***^p < 0.005.

Dimension 1 was also influenced by the dorsal/ventral processing required (but to a smaller degree) and by auditory processing (though the overall multiple regression was not significant). Therefore, we conclude that Dimension 1 best reflects the degree of concentration required to accomplish the tasks. The stimulus complexity ratings failed to reach statistical significance (*p* = 0.13). Dimension 2 was equally accounted for by dorsal/ventral processing and memory load. However, because the overall multiple regression for memory load was not significant, we consider dorsal/ventral processing to best reflect Dimension 2 (Table 3b).

In accordance with visual observations, in old participants, the MDS coordinates of Dimension 1 in the perception condition and Dimension 2 in the imagery condition seemed to be best accounted for by the dorsal/ventral distinction. In the perception condition, the MDS coordinates for Dimension 2 correlated best with the stimulus complexity ratings. In the imagery condition, Dimension 1 was best accounted for by the degree of concentration required by the tasks.

In young participants, we found that the MDS coordinates of Dimension 1 reflected dorsal/ventral processing in both perceptual and imagery conditions, while Dimension 2 reflected stimulus complexity in both perceptual and imagery conditions (Tables 3c, d).

**Table 3c:** Young participants. Multiple r and multiple regression coefficients for predicting task attributes from MDS dimensions in perception.

**Attribute**	**Multiple R**	**Dimension 1**	**Dimension 2**
Auditory	0.84”^*^	−0.75^***^	−0.54^*^
Dorsal/ventral	0.87^****^	0.997^******^	−0.02
Verbal processing	0.59	0.19	0.29
Number of stimuli	0.45	−0.22	0.22
Concentration	0.50	−0.32	−0.63
Number of processes	0.23	0.12	0.22
Hemisphere	0.37	0.02	−0.27
Visual complexity	0.33	0.10	−0.33
Verbal complexity	0.70^*^	−0.60^**^	−0.02
Stimulus complexity	0.83^****^	0.04	−0.61^*****^
Familiarity	0.71^*^	−0.58^*^	0.65^*^

Significance: ^*^ = p < 0.05, ^**^ = *p* < 0.01, ^***^ = p < 0.005, ^****^ = *p* < 0.001, ^*****^ = *p* = 0.0005, ^******^ = *p* = 0.0001.

This table has been published originally in Berardi (2025).

**Table 3d:** Young participants. Multiple r and multiple regression coefficients for predicting task attributes from MDS dimensions in imagery.

**Attribute**	**Multiple R**	**Dimension 1**	**Dimension 2**
Auditory	0.67^*^	−0.72^**^	−0.03
Dorsal/ventral	0.86^****^	0.97^*****^	−0.27
Verbal	0.56	0.27	0.17
Number of stimuli	0.54	−0.35^*^	−0.07
Concentration	0.32	−0.40	−0.09
Memory load	0.51	0.53	−0.51
Number of processes	0.21	0.14	0.18
Hemisphere	0.45	0.14	−0.29
Visual complexity	0.50	0.96	−0.31
Verbal complexity	0.60	−0.49^*^	−0.20
Stimulus complexity	78^****^	−0.30	−0.75^***^
Familiarity	0.70^*^	−0.77^**^	0.29

Significance: ^*^ = p < 0.05, ^**^ = *p* < 0.01, ^***^ = p < 0.005, ^****^ = *p* < 0.001, ^*****^ = *p* = 0.0005, ^******^ = *p* = 0.0001.

This table has been published originally in Berardi (2025).

Although the ventral tasks were generally more difficult than the spatial tasks, the MDS input was the inter-correlations among tasks rather than the response times *per se*; we can therefore exclude difficulty as an explanation for the Dimension that reflected the dorsal/ventral distinction. The correlations are, in fact, independent of the magnitude of the absolute value of the response times.

### 3.20 Between-class variance/within-class variance

Given the results of the MDS analyses, we wanted to determine whether we could objectively show that the ventral and dorsal tasks are less strongly segregated in old participants than in young participants. We therefore calculated the within-class (WCV) and between-class variance (BCV) for the MDS clusters in both perception and imagery. We did not consider mixed tasks in this analysis. If the old participants had less segregated processing pathways, we expected to find a larger between-class to within-class variance ratio in the young participants than in the old participants. The ratio of between-class (dorsal vs. ventral) to within-class variance (dorsal and ventral) was used, rather than the between-class and within-class variance *per se*, because the between-class variance can be greatly affected by the within-class variance: that is, if the within-class variance is very large, the between-class variance may also be very large, even though the overlap among tasks may be significant. The ratio, on the other hand, takes into account both types of variances simultaneously and is therefore more appropriate for comparing the degree of overlap of the two groups of tasks. We only considered for this analysis the MDS axis that best reflected the dorsal/ventral distinction.

In perception, the ratio of BCV/WCV for MDS axis 1 (which reflected dorsal/ventral processing) was 20.42 for the young participants and 6.37 for the old participants, *F*_(10, 10)_ = 3.20, N.S. The degrees of freedom for the numerator and the denominator for this and the following analyses correspond to (n – 1), where n is the number of tasks considered (11 tasks for both young and old participants). For a significance level of *p* < 0.05, *F*_(10, 10)_ must be 3.72 (see Table C9; Brown and Hollander, 1977). In imagery, the ratio of BCV/WCV for MDS axis 1 was 8.22 for young participants and 0.997 for old participants, *F*_(10, 10)_ = 8.25, *p* = 0.01. Hence, in imagery, the young participants have smaller within-group and/or larger between-group variance than the old participants. Since in old participants, MDS axis 2 mainly reflected dorsal/ventral processing, we compared the BCV/WCV of axis 1 in young participants to that of axis 2 in old participants. The ratio was 8.22 in young participants compared to only 1.19 in old participants, *F*_(10, 10)_ = 6.89, *p* < 0.01. This finding supports the inference that in imagery tasks, dorsal vs. ventral processing in older individuals is less well-segregated than in young individuals.

We also compared the BCV/WCV in MDS axis 1 for the perception and imagery conditions in the two groups separately. We found that the BCV/WCV in the perception and imagery conditions was equivalent in young participants (with ratios of 20.42 and 8.22 respectively), *F*_(10, 10)_ = 2.48, N.S. In old participants, however, the BCV/WCV was smaller in imagery than in perception (with ratios of 6.37 and 0.997, respectively), *F*_(10, 10)_ = 6.40, *p* = 0.01, indicating that old participants have less clearly segregated processing during imagery than during perception. When we compared axis 1 in perception with axis 2 in imagery in old participants, the BCV/WCV was again smaller in imagery than in perception (with ratios of 6.37 and 1.19, respectively), *F*_(10, 10)_ = 5.35, *p* = 0.01.

These results indicate that for the imagery condition, but not for the perception condition, the young participants show greater between-class/within-class variance ratios, suggesting that they indeed have more strongly segregated pathways than old participants. Another interesting point is that for old participants, but not for young ones, there was a smaller between-class variance and a larger within-class variance in the imagery condition than in the perception condition. These results indicate that dorsal and ventral tasks are equally segregated in perception and imagery for young participants but are less well-segregated in imagery than in perception for old participants.

## 4 General discussion

The results of MDS analyses indicated the presence of two major clusters reflecting processing in the dorsal and ventral pathways. However, in both perception and mental imagery, the clusters were much less well-defined in healthy old than in healthy young participants. This can be seen visually in the MDS configurations (Figures 4a–d) and was also confirmed statistically. The present results are consistent with the neuroimaging findings obtained by Grady et al. (1994), which indicated that older participants, in contrast to the young ones, had activation in the dorsal system during object processing tasks and activation in the ventral system during spatial processing tasks. In our study, old participants showed a lesser segregation of tasks that predominantly relied on processing in the dorsal and ventral pathways. This was reflected in the MDS dimensions and the generally lower correlations between the MDS dimensions and the dorsal/ventral ratings compared to those found for young participants (see Berardi, 2025). This was also reflected in the generally higher correlations between dorsal and ventral tasks than those found for young participants (see Supplementary Tables 1a–d).

Grady et al. (1992) and Grady et al. (1994) proposed that with increasing age, physiological compensatory mechanisms may occur. These mechanisms are reflected in the recruitment of additional brain areas that are originally not involved in a specific behavioral task. These physiological compensatory mechanisms may, at least for some time, enable the maintenance of equivalent behavioral performance in both young and old participants. Grady et al. (1992) and Grady et al. (1994) observed that their old participants exhibited more distributed blood flow than the young ones: the old participants recruited both dorsal and ventral areas to perform both dorsal and ventral tasks, whereas young participants only recruited ventral areas for ventral tasks and dorsal areas for dorsal tasks. Grady et al. (1992) proposed that after reaching some yet undetermined threshold, these physiological compensatory mechanisms may become inefficient, leading to impaired performance in dorsal and ventral tasks. These results have been confirmed by several other studies (e.g., Grady, 2002; Park et al., 2004; Zapparoli et al., 2019; Levine et al., 2000; Seider et al., 2021; Simmonite and Polk, 2022; Xue et al., 2023; Naspi et al., 2023; Xue et al., 2024). Our findings of reduced segregation of the dorsal and ventral visual pathways in old adults are consistent with the findings of these studies.

Moreover, although some elderly individuals can maintain cognitive performance equivalent to that of the young, others show age-related deficits (Christensen et al., 1999). Cabeza et al. (2002) compared a group of high-performing elderly to a group of low-performing elderly and a group of young participants, and using fMRI, found that the high-performing elderly showed more bilateral recruitment of the PFC in a recall and source memory task for recently studied words (see also Cabeza, 2002; Reuter-Lorenz and Lustig, 2005). They found that source memory, compared to recall, activated the right PFC in both the young and low-performing adults. However, the high-performing adults exhibited more bilateral activation of the PFC during this comparison (consistent with the ≪ HAROLD ≫ model, see Cabeza, 2002), confirming the compensatory hypothesis of brain reorganization, as opposed to the dedifferentiation hypothesis, which posits that the elderly are less able than the young to recruit specialized brain areas needed for a task (Li and Lindenberger, 1999). Grady et al. (2002) showed that age-related reductions in asymmetry can also be observed in other brain areas, particularly in the temporal and parietal regions involved in IPB tasks; using a face memory task, Grady et al. (2002) demonstrated greater bilateral asymmetry reductions in the elderly compared to the young in both temporal and parietal areas, which are presumably recruited by IPB tasks. Grady et al. (2000) also found in a PET study of a face perception task positive correlations between temporoparietal brain activity and face perception performance that were left-lateralized in young participants but bilateral for old participants. Moreover, a very interesting study by Park et al. (2004) suggested that old adults showed less specificity in the areas of the ventral visual system activated by places, faces, and chairs compared to pseudowords, whereas young participants had functionally separate peaks of activation in the ventral visual stream for places, faces, and chairs, suggesting dedifferentiation in the elderly (dedifferentiation refers to ineffective activation of usually activated brain regions by a task; see also Haupt et al., 2025).

In general, the performance of old participants was inferior to that of young participants in most dorsal and ventral tasks. Older participants were slower on all tasks, whether dorsal, ventral, or mixed, in both perceptual and imagery conditions. In perception, older participants showed a trend toward impairment in 1 out of 7 spatial tasks (brackets task) and were impaired on 2 out of 4 ventral tasks (auditory and color tasks): in all these tasks, old participants made more errors than the young. Old participants, relative to the young, also made significantly more errors in 5 spatial tasks out of 7 in the imagery condition, namely brackets, rotation, scanning, maintenance, and grids (trend), and on 1 out of 4 ventral tasks (auditory imagery). In terms of errors, two ventral tasks were preserved in both perception and imagery (object and face imagery); two dorsal tasks were also preserved (motor and spatial imagery); and the 3 mixed tasks were preserved (size, word, and tactile imagery). Therefore, our results indicate that dorsal and ventral processes are not equally affected by aging; some aspects of dorsal and ventral processing are more affected than others. These results may be explained by the specific requirements of each task and are consistent with the selective involvement of brain regions in particular types of processing.

Old participants were impaired on auditory and color tasks in perception. Old participants had more trouble judging auditory pitch than the young; this finding is consistent with studies indicating that old adults have more difficulty with high frequencies (e.g., Schieber, 1992). For the color task, they had problems judging color hue in perception, but in imagery, color judgments were preserved. Old participants also showed marginal age-related changes in the brackets task but not in the grids task. This result suggests that in perception, old participants exhibit age-related changes with metric relations but not with coordinate relations. This may indicate greater impairment in the right parietal lobe relative to the left (Kosslyn et al., 1995).

In imagery, older participants made more errors than the young on several spatial tasks: brackets, grids, rotation, scanning, and maintenance. These results suggest that old participants have a specific problem with judging spatial relations in imagery. The dissociation of the perceptual and imagery results for the spatial tasks, with relatively preserved judgment of spatial relations in perception, may be due to the fact that the stimuli in the imagery conditions were abstract. Old participants may have greater difficulty generating images of complex spatial information. This difficulty did not extend to the ventral tasks, indicating that, in general, old participants can easily retrieve images of simple objects from long-term memory. The fact that they were not impaired in the motor imagery task but were impaired in the image rotation task may support this claim: when objects are simple (such as the hands in the motor imagery task), old participants perform comparably to the young; but when they must image more complex objects (such as the blocks in the image rotation task), they are impaired (Iachini et al., 2019; Jansen et al., 2020). Alternatively, old participants may struggle with the specific processes involved in some spatial tasks: they have more difficulty than the young generating images, inspecting them, maintaining them, and rotating them. The processes involved in the rotation of objects and hands are different (Cohen et al., 1996; Kosslyn et al., 1998). Thus, the process of rotating blocks may be specifically impaired.

Our results using the IPB tasks replicate and extend previous findings. Image generation within brackets, image rotation, image maintenance, and image scanning have been evaluated previously using modifications of the tasks employed in the present study (Brown et al., 1998; Dror et al., 1993). Dror et al. (1993) only evaluated the imagery conditions of all these tasks, whereas Brown et al. (1998) assessed both the perceptual and imagery conditions of the image scanning task. Our results were generally not consistent with those obtained by Dror et al. (1993). Our results differed from theirs for the image generation brackets and image rotation tasks, in which significant age by complexity effects were obtained, which we did not replicate (although the age × complexity effect was significant for the grids task). However, when we included the 0° condition in the analyses of the rotation task, we also found a significant age × complexity effect, similar to that reported by Dror et al. (1993). Our results also differed for image scanning and image maintenance in that significant age × complexity effects were found in our study but not in theirs. Our findings are consistent with those of Brown et al. (1998), where significant age by complexity effects were found in both perception and imagery. In the present study, however, only response times, but not error rates, demonstrated significant age by complexity interactions. The differences among studies may be attributed to varying degrees of performance in the elderly participants. Greater variance in the performance of elderly participants, reflecting different underlying abilities, is one of the most consistent findings in the aging literature (e.g., Tables 1a, b for our participants). The differences among studies can likely be explained by the inclusion of elderly participants of varying abilities. However, this issue should be explored in more detail in future studies.

Generalized slowing can be excluded as a unique explanation for our findings. First, the generalized slowing hypothesis cannot account for the significant age differences we found in response times as a function of the complexity effects of the tasks (easy vs. difficult trials) or for the probe distance effects (early vs. late probes): these specific results of a disproportionate slowing of response times in the elderly compared to the young cannot be explained by general encoding or response processes. Second, logarithmic transformation of the response times in young and elderly participants showed that they still differed on most tasks, allowing us to exclude the generalized slowing hypothesis as an exclusive explanation for our findings. Third and foremost, the results we obtained align with current models of vision that categorize tasks as mediated predominantly by the dorsal visual pathway or by the ventral visual pathway. The generalized slowing hypothesis suggests that aging should have a global and uniform effect on different spatial and object tasks. Therefore, although we cannot entirely exclude its influence, our results cannot be explained exclusively by the generalized slowing hypothesis. Instead, they indicate that individual visual perceptual and mental imagery processes are selectively affected by age.

Developmental studies suggest that both the dorsal and ventral visual pathways are functional at birth, but their development in neonates, toddlers, and children is not uniform (Braddick and Atkinson, 2011; Braddick et al., 2000; Klaver et al., 2011; Lebel et al., 2008; Gilmore et al., 2018), with a more rapid development of the dorsal visual system compared to the ventral visual system during childhood. However, the ventral visual stream matures later than the dorsal visual stream due to continued gains in expertise (e.g., for faces). Regarding the development of mental imagery abilities, paradigms similar to those used in adults cannot be applied to children below 3 years, making it difficult to draw firm conclusions about development from these studies with respect to older children, adolescents, and adults. Four of the functions studied with the IPB have been assessed in children and adolescents, namely image generation, image scanning, image maintenance, and image transformation (or rotation). These functions all rely on the dorsal visual stream (Kosslyn, 1994; Berardi, 2025). The results of these studies show that children aged 3–4 years are generally unable to complete these tasks (Marmor, 1975; Kosslyn et al., 1990; Estes, 1998; Frick et al., 2013, 2014; Wimmer et al., 2015, 2016, 2017; Pedrett et al., 2023). It is only at age 5 or 6 that performance on these tasks is similar to that observed in adults, and even at this age, significant individual differences are noted (Marmor, 1975; Kosslyn et al., 1990; Estes, 1998; Funk et al., 2005; Wimmer et al., 2015, 2016, 2017; Krüger, 2018). Although children can mentally transform images at ages 5–6, mental rotation in children as young as five may depend on the characteristics of the stimuli used (e.g., Courbois, 2000). Response times in rotation tasks begin to decrease at age 5, with gradual improvement in accuracy until around age 13 (e.g., Marmor, 1975, 1977; Kail et al., 1980; Merriman et al., 1985; Hale, 1990; Snow, 1990; Kosslyn et al., 1990; Levine et al., 1999; Funk et al., 2005; Noda, 2008; Frick et al., 2009, 2013; Jansen and Kellner, 2015). The rotation of hands is also acquired by ages 5-6 and improves through adolescence (e.g., Oliveira-Souto et al., 2020; Krüger and Krist, 2009; Funk et al., 2005). To the best of our knowledge, there are fewer studies that have assessed mental imagery functions mediated by the ventral visual pathway in children and adolescents. A study using fMRI suggested that responses were already adult-like for faces, places, and objects in 3-year-olds, although these functions continue to improve through childhood and adolescence (Kamps et al., 2022). These results suggest that in children and adolescents, the dorsal visual stream may develop more rapidly, although it may be more vulnerable to psychopathology (Braddick and Atkinson, 2011), whereas in the elderly, the dorsal visual stream may be the first to be affected by age, as many studies have reported age-related changes in visual spatial tasks (e.g., Hochandel and Kaplan, 1984; Klencklen et al., 2012; Techentin et al., 2014, but see also Laurin et al., 2023).

In addition, the dorsal and ventral visual streams may be less segregated in children than in adolescents and young adults, as suggested by brain imaging or electrophysiological studies of children and adolescents (Passarotti et al., 2001, 2003; Klaver et al., 2011; Johnson et al., 2015). Interestingly, our results showed at the behavioral level less segregation of the two visual streams in the elderly compared to young adults in both perception and mental imagery. These results confirm those of previous studies in visual perception (Chen et al., 2002) and extend them to mental imagery. From the above findings, it can be suggested that the dorsal and ventral visual pathways become more segregated from childhood to adolescence, whereas in the elderly, the opposite pattern is observed, with reduced segregation of the two visual pathways compared to young adults.

The results reported in the present study are consistent with those from neuroanatomical and neurophysiological studies. Although the occipital lobes are relatively preserved with healthy aging, the parietal and temporal lobes, which are involved in dorsal and ventral processing, respectively, display neurofibrillary tangles and senile plaques (e.g., Kemper, 1984). Although healthy aging is not associated with global or regional differences in brain glucose metabolism, right–left differences in brain glucose metabolism, particularly affecting the parietal lobes, have been demonstrated (e.g., Berardi et al., 1989, 1991). Finally, fewer intercorrelations between homologous regions in the right and left hemispheres have been found in the elderly compared to young participants (Horwitz et al., 1986); it is likely that these differences have functional significance, similar to those demonstrated in this study.

### 4.1 Limitations

First, a limitation of this study was the low number of participants and the absence of a middle-aged group to provide a clearer trajectory of cognitive changes across the lifespan. Second, increasing the number of participants could enable a confirmatory factor analysis and testing of a specific model. Third, since our study did not assess brain function, we do not know whether compensatory or dedifferentiation mechanisms are at work. Compensatory mechanisms may help, at least temporarily, to maintain comparable levels of cognitive performance in old participants compared to young ones for both dorsal and ventral visual stream tasks. On the other hand, impaired performance in dorsal stream tasks may be related to inefficient compensatory mechanisms or dedifferentiation. Finally, the increased response times we observed in ventral visual stream tasks in old participants compared to young ones may be linked to dedifferentiation. Because we did not assess brain function, other studies will need to examine the precise underlying age-related brain mechanisms.

### 4.2 Future studies

Future studies combining the IPB tasks with brain imaging techniques, such as fMRI, PET, or tRMS, should determine whether performance on IPB tasks is associated with compensatory mechanisms, dedifferentiation, or neither, in the elderly brain. Longitudinal studies on healthy aging should investigate the timeframe of age-related decreases in performance across different IPB tasks and provide new insights into the time course of age-related changes. Future studies could also utilize the IPB to train visual functions to ascertain whether performance can be maintained or improved in the elderly to levels similar to those of young individuals.

### 4.3 Implications for applied contexts

The IPB battery has demonstrated its usefulness in highlighting age-related changes in various tasks predominantly mediated by the dorsal and ventral visual streams. The IPB could be employed with various groups of patients with brain lesions or other brain pathologies, such as dementia or developmental disorders, to enhance our understanding of the processes underlying visual perception and mental imagery. Moreover, the IPB could prove beneficial for assessing visual perception and mental imagery when a detailed evaluation of these functions is required, for example, in brain-damaged patients. To this end, the computerized IPB could be standardized to assess individual patients' performance on visual perceptual and mental imagery tasks. Additionally, a paper-and-pencil version of the task could be developed and standardized to assess visual perception and mental imagery in different settings with fewer material constraints than the computerized version. Finally, the IPB could be utilized for cognitive training in the elderly, in brain-damaged patients, or those with developmental disorders associated with deficits in high-level visual functions mediated by the dorsal and ventral visual pathways.

### 4.4 Conclusion

In conclusion, using MDS and principal components analysis, along with the development of experimental tasks matched for difficulty across comparable visual perceptual and mental imagery conditions, this study demonstrated a lesser segregation of the dorsal and ventral visual pathways at the behavioral level in old participants compared to young participants, in both visual perception and mental imagery.

## Data Availability

The raw data supporting the conclusions of this article will be made available by the authors, without undue reservation.
