# Supplementary material for: Age-related differences in perceptual and mental imagery abilities
Source: Front Psychol. 2026 Apr 8;16:1566776. doi: 10.3389/fpsyg.2025.1566776 (PMC13101904; doi:10.3389/fpsyg.2025.1566776)
Supplement: Supplementary file 1 [file Supplementary_file_1.pdf]

Supplementary Table 1a.
Elderly participants' correlation matrix between response times in perceptual tasks

	1.	2.	3.	4.	5.	6.	7.	8.	9.	10.	11.	12.	13.
1. Face													
2. Object	0.597												
3. Spatial	0.489	0.400											
4. Tactile	0.650	0.502	0.288										
5. Rotation	0.525	0.517	0.716	0.314									
6. Word	0.285	0.322	0.221	0.342	0.364								
7. Motor	0.597	0.504	0.653	0.382	0.627	0.225							
8. Color	0.510	0.672	0.315	0.369	0.417	0.358	0.541						
9. Maint.	0.645	0.392	0.651	0.344	0.678	0.552	0.601	0.348					
10. Auditory	0.586	0.684	0.493	0.536	0.395	0.208	0.571	0.490	0.345				
11. Grids	0.665	0.415	0.586	0.373	0.583	0.475	0.718	0.370	0.887	0.389			
12. Brackets	0.672	0.470	0.450	0.481	0.529	0.453	0.554	0.372	0.696	0.305	0.750		
13. Size	0.566	0.292	0.510	0.327	0.353	0.314	0.300	0.073	0.603	0.244	0.587	0.550	
14. Scanning	0.521	0.492	0.643	0.499	0.583	0.515	0.665	0.535	0.685	0.300	0.684	0.637	0.515

Elderly participants' correlation matrix between response times in imagery tasks

	1.	2.	3.	4.	5.	6.	7.	8.	9.	10.	11.	12.	13.
1. Face													
2. Object	0.592												
3. Spatial	0.528	0.387											
4. Tactile	0.291	0.472	0.373										
5. Rotation	0.416	0.402	0.675	0.391									
6. Word	0.421	0.528	0.145	0.211	0.384								
7. Motor	0.705	0.392	0.641	0.197	0.588	0.425							
8. Color	0.450	0.491	0.319	0.483	0.199	0.269	0.193						
9. Maint.	0.407	0.425	0.652	0.576	0.611	0.238	0.410	0.314					
10. Auditory	0.559	0.545	0.252	0.429	0.267	0.219	0.266	0.666	0.274				
11. Grids	0.341	0.458	0.296	0.245	0.391	0.443	0.549	0.239	0.280	0.495			
12. Brackets	0.534	0.441	0.381	0.120	0.500	0.352	0.601	0.182	0.392	0.443	0.768		
13. Size	0.425	0.502	0.519	0.621	0.705	0.481	0.423	0.384	0.454	0.396	0.320	0.281	
14. Scanning	0.503	0.412	0.280	0.374	0.335	0.390	0.426	0.388	0.451	0.544	0.476	0.590	0.302

Supplementary Table 1b.
Elderly participants' correlation matrix between error rates in perceptual tasks

	1.	2.	3.	4.	5.	6.	7.	8.	9.	10.	11.	12.	13.
1. Face													
2. Object	-.173												
3. Spatial	-.089	.134											
4. Tactile	.218	.168	.017										
5. Rotation	-.083	-.160	.020	.014									
6. Word	-.077	.081	-.167	.270	.657								
7. Motor	.038	.135	.366	-.315	.262	.137							
8. Color	-.137	.021	-.263	.077	.154	.344	.109						
9. Maint.	-.048	-.072	-.169	-.037	-.178	-.105	.062	-.015					
10. Auditory	.197	-.102	.031	.334	-.042	.018	-.209	.315	.003				
11. Grids	-.126	-.055	-.220	-.194	-.229	-.126	.133	.148	.511	-.202			
12. Brackets	-.086	-.067	-.046	-.189	.567	.358	-.066	.199	-.045	-.158	-.173		
13. Size	.345	-.091	.072	.328	-.099	-.296	-.341	-.402	.070	.314	-.235	.025	
14. Scanning	-.098	-.134	-.193	.277	.082	.319	-.111	.409	-.084	.251	.095	-.021	-.212

Elderly participants' correlation matrix between error rates in imagery tasks

	1.	2.	3.	4.	5.	6.	7.	8.	9.	10.	11.	12.	13.
1. Face													
2. Object	-.011												
3. Spatial	.125	.284											
4. Tactile	.172	.340	-.317										
5. Rotation	.532	.116	.194	.196									
6. Word	.307	.030	.233	-.067	.041								
7. Motor	-.022	-.179	.253	-.215	-.096	.052							
8. Color	.006	.379	.135	-.052	.038	.026	-.300						
9. Maint.	.468	.288	.124	-.007	.403	.122	.057	.238					
10. Auditory	.044	.243	.385	-.119	.057	.065	-.313	.115	-.048				
11. Grids	.188	.273	.054	.219	.019	.200	-.149	-.046	.325	.148			
12. Brackets	.310	.065	.227	.239	.332	.214	.255	-.020	.354	-.210	.076		
13. Size	.470	.051	.172	-.283	.475	.204	.038	.181	.530	-.001	.076	.145	
14. Scanning	.152	.160	.458	-.036	.354	.027	.038	.075	.029	.268	-.279	.137	.010

Supplementary Table 1c.
Young participants' correlation matrix between response times in perceptual tasks

	1.	2.	3.	4.	5.	6.	7.	8.	9.	10.	11.	12.	13.
**1.** Face													
2. Object	0.462												
3. Spatial	-0.064	0.151											
4. Tactile	0.437	0.255	0.076										
5. Rotation	0.216	0.342	0.111	0.101									
6. Word	-0.123	0.032	0.227	0.388	0.273								
7. Motor	0.180	0.184	0.397	0.151	0.585	0.545							
8. Color	0.659	0.638	0.199	0.249	0.233	-0.090	0.171						
9. Maint.	0.179	0.298	0.428	0.153	0.462	0.535	0.688	0.294					
10. Auditory	0.462	0.479	0.134	0.278	0.194	-0.127	0.131	0.451	0.302				
11. Grids	0.259	0.267	0.335	0.422	0.491	0.576	0.609	0.210	0.791	0.359			
12. Brackets	0.232	0.234	0.094	0.484	0.320	0.602	0.440	0.148	0.381	0.208	0.620		
13. Size	0.494	0.221	0.045	0.615	0.331	0.388	0.378	0.203	0.382	0.413	0.605	0.678	
14. Scanning	0.079	0.236	0.130	0.187	0.568	0.385	0.397	0.163	0.477	0.027	0.353	0.222	0.259

Young participants' correlation matrix between response times in imagery tasks

	1.	2.	3.	4.	5.	6.	7.	8.	9.	10.	11.	12.	13.
**1.** Face													
2. Object	0.212												
3. Spatial	0.089	-0.001											
4. Tactile	0.475	0.088	0.335										
5. Rotation	0.159	0.443	0.257	-0.127									
6. Word	-0.019	0.366	0.291	0.112	0.017								
7. Motor	0.407	0.057	0.603	-0.035	0.529	-0.072							
8. Color	0.022	0.113	0.129	-0.053	0.067	-0.037	0.109						
9. Maint.	-0.052	0.267	0.507	-0.023	0.284	0.578	0.271	-0.209					
10. Auditory	0.456	0.449	0.232	0.302	0.177	0.373	0.156	0.419	0.251				
11. Grids	-0.117	-0.008	0.435	0.078	0.349	0.434	0.232	0.075	0.512	0.128			
12. Brackets	0.098	0.238	0.540	0.241	0.506	0.337	0.348	0.069	0.358	0.275	0.722		
13. Size	0.446	0.517	0.261	0.290	0.351	0.341	0.288	0.300	0.344	0.845	0.190	0.420	
14. Scanning	0.165	0.301	0.404	0.059	0.475	0.336	0.431	-0.076	0.386	0.075	0.414	0.605	0.177

Supplementary Table 1d.
Young participants' correlation matrix between error rates in perceptual tasks

	1.	2.	3.	4.	5.	6.	7.	8.	9.	10.	11.	12.	13.
1. Face													
2. Object	0.030												
3. Spatial	0.168	-0.012											
4. Tactile	0.156	0.127	-0.095										
5. Rotation	0.191	0.112	0.030	0.499									
6. Word	0.360	0.062	-0.083	0.213	0.255								
7. Motor	-0.150	0.097	-0.131	-0.026	-0.187	-0.190							
8.Color	0.046	-0.037	0.362	0.097	0.017	-0.254	0.091						
9. Maint.	0.219	-0.042	-0.146	-0.145	-0.104	0.285	-0.269	-0.200					
10. Auditory	0.081	0.190	0.034	-0.026	0.210	-0.103	0.033	0.256	-0.142				
11. Grids	-0.024	-0.066	-0.213	-0.162	-0.126	-0.161	0.263	0.195	-0.043	0.252			
12. Brackets	0.250	0.208	-0.115	0.130	0.253	0.099	-0.161	0.173	0.238	0.086	-0.250		
13. Size	-0.172	0.104	-0.070	-0.029	0.200	-0.169	-0.029	0.132	-0.120	0.211	-0.152	0.397	
14. Scanning	0.115	-0.049	-0.168	0.084	0.000	0.329	-0.207	-0.231	0.433	-0.164	-0.248	0.152	0.000

Young participants' correlation matrix between error rates in imagery tasks

	1.	2.	3.	4.	5.	6.	7.	8.	9.	10.	11.	12.	13.
1. Face													
2. Object	-0.204												
3. Spatial	0.149	0.227											
4. Tactile	0.177	-0.136	-0.060										
5. Rotation	-0.155	0.369	0.093	-0.133									
6. Word	0.185	0.229	0.359	-0.222	0.476								
7. Motor	0.139	0.227	0.450	0.076	0.245	0.312							
8. Color	-0.016	0.093	-0.092	0.174	0.126	-0.014	-0.117						
9. Maint.	0.234	0.029	-0.189	0.159	0.154	-0.023	-0.065	0.406					
10. Auditory	-0.113	-0.071	-0.272	0.105	-0.233	-0.192	-0.091	-0.067	-0.007				
11. Grids	0.009	0.068	0.366	0.167	0.028	0.229	0.297	-0.116	-0.171	-0.457			
12. Brackets	0.165	-0.009	0.048	0.022	0.190	0.097	0.081	-0.239	0.118	-0.259	0.271		
13. Size	0.207	0.393	0.302	-0.262	0.212	0.437	0.398	-0.043	0.035	-0.130	0.098	0.599	
14. Scanning	0.079	0.152	0.260	0.037	0.445	0.327	0.273	0.202	-0.090	-0.328	0.212	0.361	0.336

Note : Tables 1c and 1d have been originally published in Berardi AM (2025). Varieties of imagery and perception: the structure of task differences. Front. Psychol. 16:1568148.
doi: 10.3389/fpsyg.2025.1568148
